# Derepression of transposable elements in the mouse prefrontal cortex disrupts social behavior

**DOI:** 10.1073/pnas.2510663122

**Published:** 2025-12-17

**Authors:** R. Kijoon Kim, Corinne Smith, Natalie L. Truby, Shelbey R. Strandberg, Jessica L. Bell, Nic Carwile, Gabriella M. Silva, Rachael L. Neve, Theingi Aung, Xiaohong Cui, Peter J. Hamilton

**Affiliations:** ^a^Department of Neuroscience and Anatomy, Virginia Commonwealth University School of Medicine, Richmond, VA 23298; ^b^Gene Delivery Technology Core, Massachusetts General Hospital, Boston, MA 02145

**Keywords:** transposable elements, TRIM28, social behavior, prefrontal cortex

## Abstract

The neurobiological origins of social behaviors are poorly understood. Previous studies have linked the function of a single Krüppel-associated box zinc finger protein (KZFP), ZFP189, in the mouse prefrontal cortex (PFC), with the regulation of transposable elements (TEs), immune genes, and social behaviors. Here, we expand the scope of inquiry to explore the relationship between collective PFC KZFP function and social behaviors by altering the function of the cognate KZFP interacting protein TRIM28 within the PFC of male and female mice. We reprogrammed natural TRIM28^WT^ by replacing the endogenous, transcriptionally repressive domain with a synthetic, enhanced transcriptional activation domain VP64-p65-Rta (TRIM28^VPR^) or by excising the transcriptional regulatory domain (TRIM28^NFD^). Upon intra-PFC viral-mediated delivery of TRIM28 variants, we observed that inversion of TRIM28 transcriptional control via HSV-TRIM28^VPR^ selectively produced deficits in social behaviors, without affecting nonsocial behaviors. RNA sequencing of manipulated PFC revealed that HSV-TRIM28^VPR^ drove transcriptional escape of all classes of TEs, particularly those located within intronic and enhancer regions proximal to downregulated immune genes. HSV-TRIM28^VPR^-mediated social deficits were reversible by intra-PFC repletion of interferon cytokines. These data point to PFC KZFP–TRIM28 interactions as necessary to stabilize genomic TEs to enable *cis*-regulation of key immune gene expression, which enhances organismal capacity for complex, prosocial behaviors.

## Significance

Social behavior is essential to quality of life but is often impaired in neurodevelopmental and neuropsychiatric disorders. At the genetic level, the origins of complex social behaviors are not well understood. Here, we developed molecular tools to demonstrate a direct mechanistic link between the release of transposable elements in the mouse prefrontal cortex, decreased interferon-related gene expression, and impairments in group-based social behaviors. These findings suggest that loss of transcriptional repression of transposable elements impairs *cis*-regulation of immune genes, which leads to a specific disruption of social behaviors. Collectively, this work provides mechanistic insight into the molecular origins of social behaviors and demonstrates that the maintained transcriptional control of brain transposable elements is necessary for prosocial behaviors.

Social behaviors are a fundamental aspect of group living and are central to the health of both the individual and society (1–3). Alterations to social behaviors are associated with many brain disorders. For example, social withdrawal characterizes conditions including major depressive disorder, autism spectrum disorder (ASD), and schizophrenia, while hypersociability occurs in Angelman and Williams syndromes (4). However, the neurobiological mechanisms that enable complex social behaviors, and how these mechanisms can go awry, are incompletely understood.

Since their discovery by Barbara McClintock, genomic transposable elements (TEs), or self-replicating DNA sequences, have been identified as molecular drivers capable of altering genome size and stability and are thought to contribute to the diversification and evolution of species (5–8). However, due to the potential deleterious effects of active transposition of TEs, maintaining a transcriptionally silenced TE landscape is vital to the health of host organisms (9–13). Genomic regions consisting of transcriptionally silenced TEs have been co-opted by their hosts in diverse ways, including in formation of *cis*-regulatory DNA regions (14–16) and by contributing to the regulation of the adaptive immune system (17–19). Recently, several groups have begun to identify evidence that links social and other complex behaviors with proper regulation of these TE-associated immune responses in the brain (20–22).

Krüppel-associated box (KRAB) zinc finger proteins (KZFP) comprise an evolutionarily ancient and diverse family of transcription factors that have evolved to limit TE transposition by repressing TE expression via the recruitment of tripartite motif protein 28 (TRIM28), also called KAP1 (23–27). KZFP-conserved KRAB domains recruit TRIM28, and TRIM28 recruits histone methyltransferases via the TRIM28 HP1, PHD, and BROMO domains (28, 29). This mechanism contributes to the formation of heterochromatin at KZFP-DNA-binding sites and transcriptional silencing of KZFP gene targets, including TEs (30).

Here, we developed novel TRIM28 variants delivered via herpes simplex virus (HSV) to the prefrontal cortex (PFC) of male and female mice to analyze the transcriptional and behavioral consequences of TRIM28-mediated transcriptional function and dysfunction in the brain. In addition to the endogenous repressive TRIM28^WT^ protein, we created TRIM28^NFD^, which retains the KZFP-binding domain but lacks the transcriptionally repressive HP1, PHD, and BROMO domains, as well as TRIM28^VPR^, which replaces these transcriptionally repressive domains with the transcriptional activator VP64-p65-Rta (VPR). We conducted a battery of behavioral and transcriptomic assays to determine the behavioral and molecular consequences of dysregulating TRIM28 transcriptional control in PFC. We observed that intra-PFC delivery of HSV-TRIM28^VPR^ altered social behaviors, characterized by a lack of interest in novel social interactions and impaired ability to engage in previously established social hierarchies, without producing detectable nonsocial behavioral changes. In RNA sequencing (RNAseq) analyses, HSV-TRIM28^VPR^ drove TE escape and downregulation of proximal immune genes. Ablation of TRIM28-mediated transcription through delivery of HSV-TRIM28^NFD^ induced similar, but modest social behavioral deficits and transcriptional changes, pointing to a graded relationship between magnitude of PFC TE dysregulation and degree of social deficits. These social deficits could be restored by introduction of exogenous immune factors to manipulated PFC. Collectively, this work resolves a mechanistic relationship between cortical TEs, immune functions, and social cognition.

## Results

### TRIM28 Variants Bidirectionally Control the Expression of a KZFP-Regulated Gene In Vitro.

**Fig. 1.**
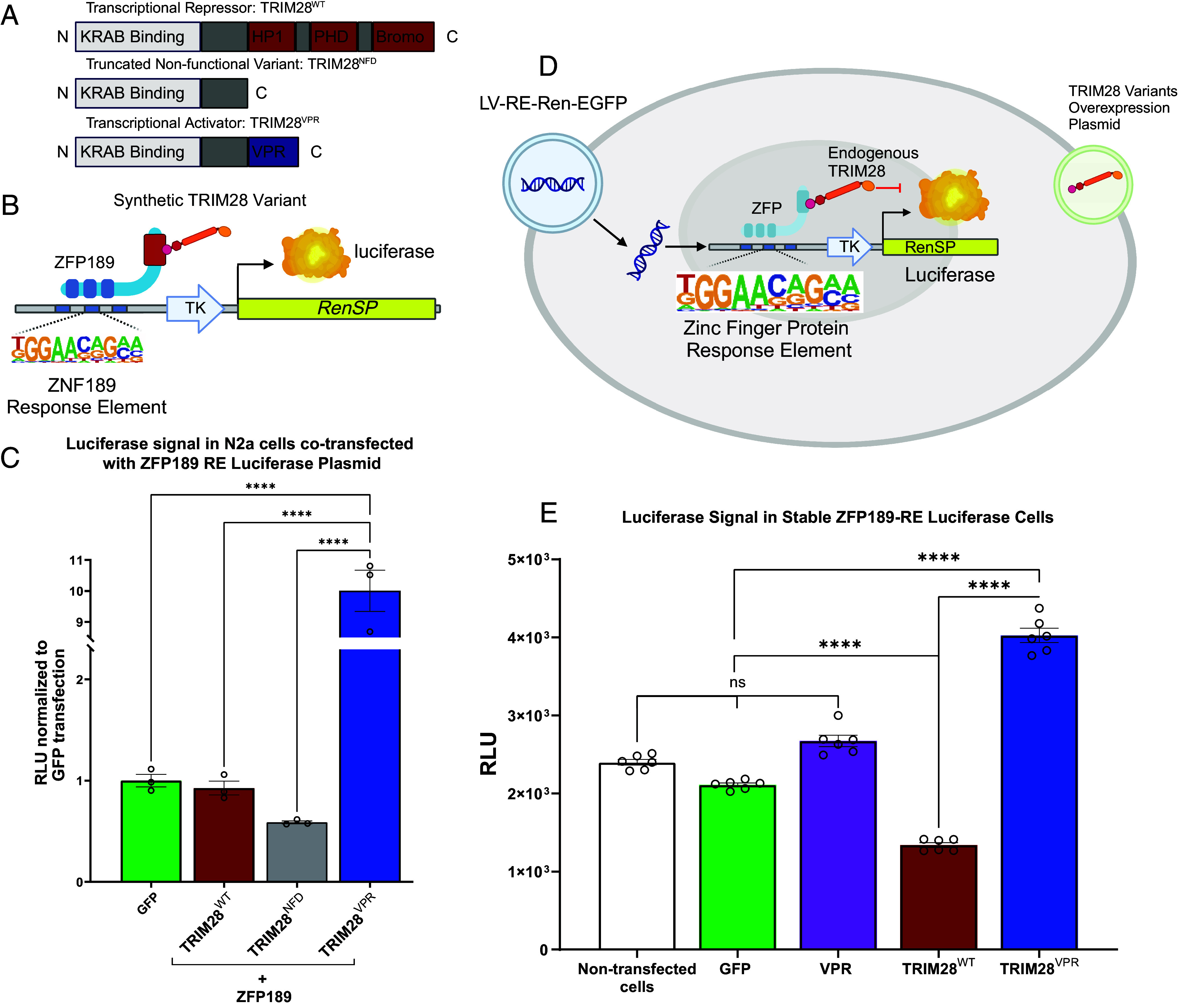
TRIM28 variants bidirectionally control the expression of a KZFP-regulated gene. (*A*) Schematic of novel TRIM28 variant proteins. (*B*) Schematic of the luciferase reporter, which contains the KZFP ZFP189 REs upstream of a TK promoter and RenSP LightSwitch™ luciferase gene. (*C*) Cotransfection of Neuro2a (N2a) cells with separate overexpression vectors containing the luciferase reporter, ZFP189 (a representative KZFP), and a TRIM28 variant shows TRIM28^VPR^ significantly upregulates luciferase signal (*P* < 0.0001, one-way ANOVA with Bonferroni correction, n = 3 per group). (*D*) Schematic of a stable N2a cell line that stably expresses the luciferase reporter (LV-RE-Ren-EGFP-N2a). These stable cells were transfected with a single overexpression vector containing TRIM28 variants. (*E*) TRIM28^VPR^ significantly increases luciferase signal, while TRIM28^WT^ significantly decreases luciferase signal, compared to nontransfected control cells, or cells transfected with overexpression vectors for GFP or an isolated VPR domain (TRIM28^VPR^: *P* < 0.0001 versus nontransfected cells, GFP, and VPR. TRIM28^WT^: *P* < 0.0001 versus nontransfected cells, GFP, and VPR. One-way ANOVA with Bonferroni correction, n = 6 per group).

We cloned three TRIM28 variants into overexpression plasmids: TRIM28^WT^, identical to the endogenous TRIM28 protein containing a N-terminal KRAB-binding domain and C-terminal HP1, PHD, and BROMO domains; TRIM28^NFD^ with these C-terminal domains excised; and TRIM28^VPR^ with these C-terminal domains replaced by the synthetic transcriptional activator VPR (Fig. 1*A*). We validated that our TRIM28 constructs are translated into protein and do not alter endogenous TRIM28 protein expression, both in vitro and in vivo (*SI Appendix*, Fig. S1). Next, we applied a modified luciferase transfection assay that our group has previously implemented (22). In cultured Neuro2a cells, we simultaneously cotransfected 1) a *luciferase* reporter plasmid under the control of a thymidine kinase (TK) promoter and possessing multiple ZFP189-recruiting DNA response elements (REs); 2) a plasmid overexpressing ZFP189, a representative KZFP that has previously been demonstrated to regulate this *luciferase* reporter gene (22); and 3) a plasmid overexpressing one of our TRIM28 variants or a GFP plasmid control (Fig. 1*B*). Luciferase signal was calculated as relative light units (RLUs) as a proxy for gene transcription (Fig. 1*C*). We saw that TRIM28^VPR^ increased luciferase signal (*P* < 0.0001, one-way ANOVA with Bonferroni correction), indicating that it acts as a gene activator at KZFP-regulated genes.

We next developed a Neuro2a cell line stably expressing the ZFP189-sensitive *luciferase* reporter (Fig. 1*D*). By transfecting these stable cells with TRIM28 variants, we observed that TRIM28^WT^ decreased luciferase signal compared to nontransfected, GFP, or isolated VPR domain controls (*P* < 0.0001 for all). Conversely, we observed that TRIM28^VPR^ increased luciferase signal compared to these controls (*P* < 0.0001 for all), indicating that TRIM28^WT^ and TRIM28^VPR^ produce opposite effects on KZFP-regulated gene transcription (Fig. 1*E*). Collectively, these in vitro studies indicate our synthetic TRIM28 variants can, as designed, specifically up- or down-regulate the expression of KZFP-regulated genes.

### Inverting TRIM28-Mediated Transcriptional Control in the Prefrontal Cortex Causes Social Behavioral Deficits in Male and Female Mice.

**Fig. 2.**
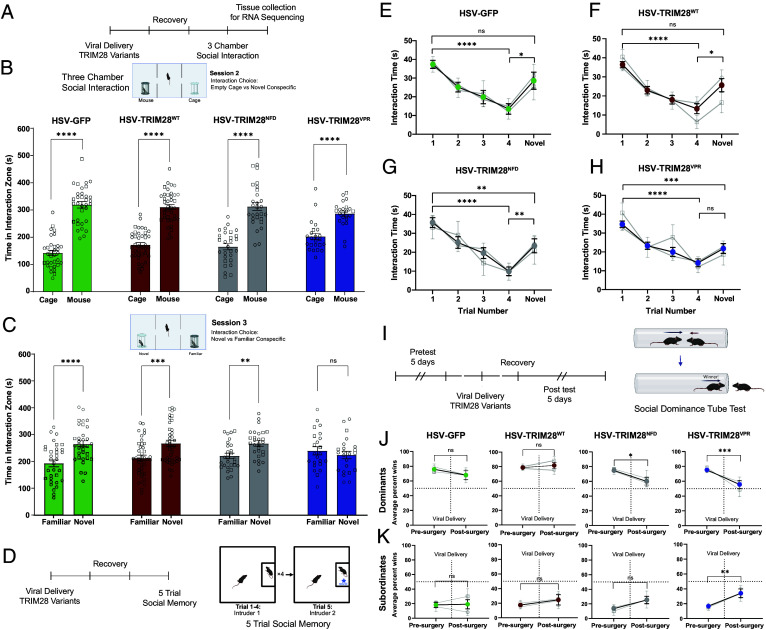
Disrupting PFC TRIM28 function disrupts social behavior. (*A*) Timeline of three-chamber social interaction and prefrontal cortex (PFC) tissue collection. Each vertical hash is a day (HSV-GFP: n = 10 females, 23 males; HSV-TRIM28^WT^: n = 10 females, 29 males; HSV-TRIM28^NFD^: n = 10 females, 18 males; HSV-TRIM28^VPR^: n = 6 females, 17 males). (*B*) In Session 2 of three-chamber social interaction (cage v. mouse), all viral conditions spend more time interacting with a novel conspecific than an empty cage (Interaction term *P* = 0.0012; all viral treatments Cage v. Mouse *P* < 0.0001, two-way ANOVA with Bonferroni correction). (*C*) In Session 3 of three-chamber social interaction (familiar v. novel), HSV-TRIM28^VPR^ ablates the preference for socializing with a novel conspecific over a familiar one (Interaction term *P* = 0.0057; HSV-TRIM28^VPR^ Familiar v. Novel, *P* > 0.05, two-way ANOVA with Bonferroni correction), while this preference is preserved in HSV-GFP, HSV-TRIM28^WT^, and HSV-TRIM28^NFD^-treated mice (*P* < 0.0001, 0.0003, 0.0098, respectively, two-way mixed effects ANOVA with Bonferroni correction). (*D*) Timeline and experimental design for five-trial social memory (HSV-GFP: n = 8 females, 11 males; HSV-TRIM28^WT^: n = 8 females, 19 males; HSV-TRIM28^NFD^: n = 8 females, 19 males; HSV-TRIM28^VPR^: n = 8 females, 27 males). (*E*–*G*) Mice receiving HSV-GFP, HSV-TRIM28^WT^, or HSV-TRIM28^NFD^ habituate to a repeated social partner and renew interest for a novel partner (Interaction term *P* = 0.8957; Trial 1 v. 4: *P* < 0.0001 for all, Trial 4 v. novel: *P* = 0.0123, 0.0309, 0.0024, respectively, two-way ANOVA with Bonferroni correction). Only HSV-TRIM28^NFD^-treated mice spend significantly less time interacting with the novel interaction target than with the initial interaction target (Trial 1 v. novel, *P* = 0.0098, two-way ANOVA with Bonferroni correction). (*H*) Mice receiving HSV-TRIM28^VPR^ habituate to a repeated social partner, but do not renew interest for a novel partner (Trial 1 v. 4: *P* < 0.0001, Trial 4 v. novel, *P* > 0.05, two-way ANOVA with Bonferroni correction). These mice also spend significantly less time interacting with the novel interaction target than with the initial interaction target (Trial 1 v. novel, *P* = 0.0003, two-way ANOVA with Bonferroni correction). (*I*) Timeline and experimental design for social dominance tube test. (*J*) Comparing average pretest win rate to average posttest win rate, dominant mice who received HSV-TRIM28^NFD^ (n = 6 females, 11 males) and -TRIM28^VPR^ (n = 6 females, 12 males) had significantly decreased win rates in the posttest (Interaction term *P* = 0.0174; *P* = 0.0092 and *P* = 0.0002, respectively, two-way ANOVA with Bonferroni correction). Receiving HSV-GFP (n = 6 females, 6 males) or -TRIM28^WT^ (n = 6 females, 6 males) did not significantly alter dominant mouse win rates (*P* > 0.05, two-way ANOVA with Bonferroni correction). (*K*) Comparing average pretest win rate to average posttest win rate, subordinate mice who received HSV-GFP (n = 6 females, 6 males), -TRIM28^WT^ (n = 6 females, 6 males), or -TRIM28^NFD^ (n = 6 females, 11 males) did not have significantly different win rates (Interaction term *P* = 0.3414; *P* > 0.05, two-way ANOVA with Bonferroni correction), while subordinate mice who received HSV-TRIM28^VPR^ (n = 6 females, 12 males) had a significant increase in win rate (*P* = 0.0099, two-way ANOVA with Bonferroni correction). Squares indicate females; circles indicate males. Gray lines show male and female data separated; black lines show both sexes pooled.

We next sought to characterize the in vivo behavioral consequences of dysregulating TRIM28-mediated transcriptional control in the PFC of male and female mice. We virally delivered our HSV-packaged TRIM28 variants or GFP control via stereotaxic surgery to the PFC (31, 32) and, in distinct experimental cohorts, subjected test mice to social behavioral assays (Fig. 2 *A*, *D*, and *I*). As expected in a three-chamber social interaction task, when presented with choice between a sex- and age-matched conspecific or an empty cage, all mice displayed a normal preference for social interaction (*P* < 0.0001 for all) (Fig. 2*B*). Notably, when presented with a novel versus familiar conspecific, all except HSV-TRIM28^VPR^ mice showed normal preference to interact with the novel conspecific (*P* < 0.0001, 0.0003, 0.0098, respectively) (Fig. 2*C*). Yet, mice receiving PFC HSV-TRIM28^VPR^ specifically did not show this preference for interacting with a novel sex- and age-matched conspecific over a familiar conspecific (*P* > 0.05) (Fig. 2*C*). This lack of preference for novel social interaction cannot be explained by alterations in anxiety or locomotor phenotypes, as neither HSV-TRIM28^VPR^ nor any other treatment group displayed alterations in elevated plus maze exploration, novelty suppressed feeding, or sucrose preference test (*SI Appendix*, Fig. S2).

We next aimed to test whether this social preference phenotype could be explained by deficits in social recognition. We performed a five-trial social memory test (Fig. 2*D*), where experimental mice were subjected to four sequential exposure to the same age- and sex-matched conspecific, followed by a fifth session with a novel conspecific (22, 33). Typically, mice will spend decreasing amounts of time interacting with the same conspecific through sessions 1–4, and renew social interaction behaviors during session 5. Mice receiving HSV-TRIM28^WT^, -TRIM28^NFD^, and -GFP all displayed this characteristic pattern of behavior (Fig. 2 *E*–*H*). However, mice receiving HSV-TRIM28^VPR^ did not show a significantly renewed interest in interacting with the novel mouse in session 5, suggesting the salience of novel social interaction with the new conspecific was diminished (*P* > 0.05, two-way ANOVA with Bonferroni correction) (Fig. 2*H*). Yet, the progressive decrease in interaction time through sessions 1–4 for the HSV-TRIM28^VPR^ mice does suggest that the capacity for social memory is intact (*P* < 0.0001).

To more completely characterize the nature of TRIM28^VPR^-mediated social behavioral deficits, we performed repeated social dominance tube tests (Fig. 2*I*). Our group has previously employed this task to characterize how viral delivery of transcription factor constructs affects awareness and participation in social hierarchies (22). Conventionally, dominance is defined as consistently winning the majority of tube tests against cage mates, while subordinance reflects consistent losses against cage mates (34, 35). Here, we allowed cages of five sex-matched mice to establish a social hierarchy, then delivered HSV-GFP, -TRIM28^WT^, -TRIM28^NFD^, or -TRIM28^VPR^ to the PFC of both dominant and subordinate mice, and retested social hierarchy for 5 d postsurgery. Intermediate ranked mice received intra-PFC HSV-GFP to match surgical experience to the test mice. We analyzed results pre- versus postsurgery between viral conditions separately between dominant mice and subordinate mice.

As expected, dominant and subordinate mice receiving HSV-GFP and HSV-TRIM28^WT^ remained dominant and subordinate, respectively, with win rates remaining significantly different from random chance postsurgery (Dominants: *P* = 0.0171, 0.0010, respectively, Subordinates: *P* = 0.0112. 0.0107, respectively, Wilcoxon signed-rank test) (Fig. 2 *J* and *K*). Mice receiving HSV-TRIM28^NFD^ did not completely maintain their social hierarchy positions postsurgery, as dominant mice became significantly less dominant (*P* = 0.0092, two-way ANOVA with Bonferroni correction), though they still won more often than random chance (*P* = 0.0215, Wilcoxon signed-rank test). Yet, subordinate mice receiving HSV-TRIM28^NFD^ remained similarly subordinate (*P* > 0.05, two-way ANOVA with Bonferroni correction) and maintained a lesser win rate compared against random chance, indicating the maintained awareness of their hierarchical position (*P* = 0.0366, Wilcoxon signed-rank test) (Fig. 2 *J* and *K*). However, HSV-TRIM28^VPR^ caused both dominant and subordinate mice to revert toward a random chance of winning (*P* > 0.05, Wilcoxon signed-rank test) (Fig. 2 *J* and *K*). Further, dominant and subordinate mice receiving HSV-TRIM28^VPR^ all performed significantly differently postsurgery compared to their presurgery hierarchical position (*P* = 0.0002, 0.0099, respectively, two-way ANOVA with Bonferroni correction). This indicates inversion of PFC TRIM28 molecular function with TRIM28^VPR^ is sufficient to remove the mouse’s capacity to participate in social hierarchies and/or remove their capacity for performing cooperative social strategies required to consistently resolve a social conflict, here modeled in a head-to-head tube test.

Finally, we tested novel object recognition to determine whether this deficit in novel social interaction could be explained by deficits in recognition of nonsocial stimuli. We applied a previously validated Y-maze novel object recognition paradigm (36, 37) and found mice in all treatment groups displayed identical performance on the Y-maze spontaneous alternation task as well as retained ability to recognize novel objects (*SI Appendix*, Fig. S3). These data suggest that HSV-TRIM28^VPR^ uniquely disrupts aspects of social behaviors, but not other cognitive behaviors that rely on general nonsocial recognition.

### TRIM28^VPR^ Derepresses Transposable Elements in the Prefrontal Cortex.

**Fig. 3.**
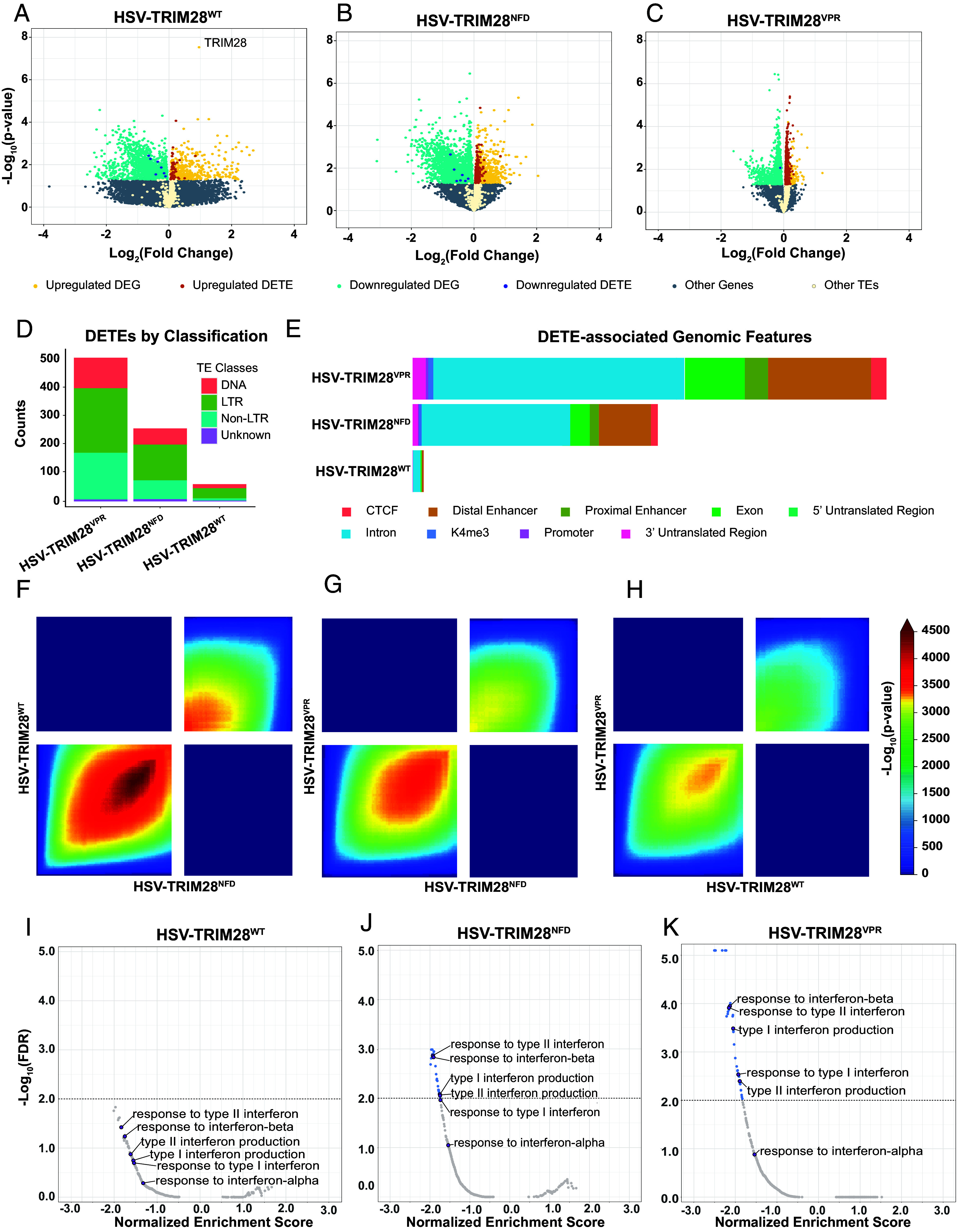
Disrupting TRIM28 function drives the release of transposable elements and impairs immune gene expression in PFC. (*A*–*C*) Volcano plots of all gene and TE transcripts affected by HSV-TRIM28^VPR^, HSV-TRIM28^NFD^, and HSV-TRIM28^WT^ normalized to the GFP control condition (HSV-GFP: 9 females, 9 males; HSV-TRIM28^WT^: 10 females, 10 males; HSV-TRIM28^NFD^: 8 females, 9 males; HSV-TRIM28^VPR^: 10 females, 9 males; all analysis performed on data from combined sexes). DEGs and DETEs were considered statistically significant if *P* < 0.05. HSV-TRIM28^VPR^ induced many more upregulated DETEs compared to other TRIM28 variants. (*D*) HSV-TRIM28^VPR^ and HSV-TRIM28^NFD^ induced activation of all classes of TEs, and TEs of the non-LTR class were disproportionately activated (Chi-square goodness of fit test, *P* < 0.0001 for both). (*E*) HSV-TRIM28^VPR^ and HSV-TRIM28^NFD^ activated TEs that were predominantly derived from intronic and distal enhancer genomic features. (*F*–*H*) Rank–rank hypergeometric overlap graphs demonstrate threshold-free comparisons between gene lists. Gene expression profiles induced by HSV-TRIM28^VPR^ are more similar to HSV-TRIM28^NFD^ than HSV-TRIM28^WT^. The *Lower Left* quadrant represents coupregulation, and the *Upper Right* quadrant represents codownregulation. (*I*–*K*) Volcano plots showing gene set enrichment analysis terms generated from all DEGs in HSV-TRIM28^WT^, -TRIM28^NFD^, and -TRIM28^VPR^. Interferon-related gene ontology terms are specifically labeled. A significance cutoff of FDR < 0.01 was applied. Interferon-related ontology terms were significantly downregulated by HSV-TRIM28^VPR^ to a greater extent than HSV-TRIM28^NFD^ and were not affected by HSV-TRIM28^WT^.

In order to understand the TRIM28-driven brain molecular mechanisms associated with the observed social deficits, we performed bulk RNAseq at day four on manipulated PFC tissue from individual male and female mice. This timeline was selected to reflect the timing of emergence of the behavioral changes observed above (Fig. 2). Given the growing body of literature linking KZFPs to silencing mechanisms of TEs (22, 23, 26, 28, 38), we hypothesized that impairment of TRIM28 gene-regulatory function would lead to widespread dysregulation of TEs. Thus, we utilized a modified RNAseq analytical pipeline that included analyses for TEs, which are typically excluded in RNAseq analyses due to multiple read alignment (39, 40). All differentially expressed genes (DEGs) and differentially expressed TEs (DETEs) were calculated relative to the HSV-GFP viral control condition. We observed that HSV-TRIM28^WT^ produced up- and down-regulation of DEGs, and, as expected, did not produce many DETEs compared to the GFP control (Fig. 3*A*; full DEG and DETE gene list in Dataset S1). In contrast, HSV-TRIM28^NFD^ produced more upregulated DETEs (Fig. 3*B*; full DEG and DETE gene list in Dataset S2), pointing to dominant negative function of TRIM28^NFD^ capable of competing with endogenous PFC TRIM28 and derepressing TEs. Most strikingly, HSV-TRIM28^VPR^ asymmetrically drove robust upregulation of DETEs (Fig. 3*C*; full DEG and DETE gene list in Dataset S3), indicating that the additional gene-activating function of the VPR moiety on TRIM28^VPR^ serves to actively drive TE escape.

We next assessed the subcategories of DETEs dysregulated by each of our synthetic TRIM28 variants. Irrespective of direction of regulation, HSV-TRIM28^WT^ produced 61 DETEs compared to the GFP control, while HSV-TRIM28^NFD^ produced 259 DETEs and HSV-TRIM28^VPR^ produced 510 DETEs (Fig. 3*D*). The distribution of LTR, non-LTR, and DNA transposons differed across viral treatment conditions (*SI Appendix*, Table S1). In all TRIM28 variant conditions, TEs in the LTR class account for approximately half, and DNA transposons account for approximately 20% of all DETEs detected. However, non-LTR class TEs expanded from 13% of DETEs in HSV-TRIM28^WT^ to 26% of DETEs in HSV-TRIM28^NFD^ and 33% of DETEs in HSV-TRIM28^VPR^, indicating the non-LTR class of TEs were disproportionately derepressed by our synthetic TRIM28 variants compared to HSV-TRIM28^WT^ (Chi-square goodness of fit, *P* < 0.0001 for HSV-TRIM28^NFD^ and HSV-TRIM28^VPR^). Additionally, we annotated the genomic features with which these DETEs overlap. All three TRIM28 variants affected DETEs originating from intronic and distal enhancer genomic regions, accounting for approximately 80% of all annotated genomic features (Fig. 3*E* and *SI Appendix*, Table S2). These transcriptomic data suggest that inverted TRIM28 function in PFC derepresses primarily non-LTR TEs residing within genomic distal enhancer and intronic regions.

### Derepression of Transposable Elements Downregulates Immune Genes.

We next sought to explore the differences in canonical gene transcription produced by our TRIM28 variants. We first applied rank–rank hypergeometric overlap (RRHO) analyses to assess the broad congruence of gene-expression patterns. RRHO captures threshold-free similarities and differences across gene lists (41). Using the RRHO2 package, we observed that the collective gene expression profile affected by HSV-TRIM28^VPR^ was more similar to HSV-TRIM28^NFD^ than to HSV-TRIM28^WT^ (Fig. 3 *G* and *H*). Further, HSV-TRIM28^NFD^ produced an intermediate transcriptional profile that was more broadly similar to HSV-TRIM28^WT^ than to HSV-TRIM28^VPR^ (Fig. 3 *F* and *G*), pointing to a gradation in the concordance of PFC transcriptional profile that is stratified across TRIM28 normal function (HSV-TRIM28^WT^), nonfunction (HSV-TRIM28^NFD^), and anti-function (HSV-TRIM28^VPR^). This also mirrors the gradation of behavioral effects observed in Fig. 2.

To characterize the nature of affected genes in each condition, we performed gene set enrichment analysis (GSEA) of total DEGs produced by each TRIM28 variant (Fig. 3 *I*–*K*). To improve specificity of ontology terms while preserving an exploratory approach, the maximum number of gene IDs per category was limited to 300, and only terms with a false discovery rate (FDR) < 0.01 were designated significant. In the GSEA, which accounts for both the directionality and magnitude of expression change, we observed that while all three TRIM28 variants primarily display downregulated gene ontology terms, the magnitude of this downregulation mirrors the gradation of effect observed in both TE upregulation (Fig. 3 *A*–*C*) and behavioral dysregulation (Fig. 2 *J* and *K*). In particular, we observed that interferon-related terms adhered to the same pattern of downregulation, with HSV-TRIM28^WT^ inducing a nonsignificant downregulation (Fig. 3*I*), HSV-TRIM28^NFD^ a moderate but significant downregulation (Fig. 3*J*), and HSV-TRIM28^VPR^ the greatest and significant downregulation (Fig. 3*K*). This is recapitulated in the expression levels of specific interferon genes (*SI Appendix*, Fig. S4). Accounting for the growing body of literature linking PFC immune functions to social behaviors (21, 42, 43), including from our group (22), this impact to immune gene expression represents a potential molecular mechanism that explains the HSV-TRIM28^VPR^-driven social deficits.

**Fig. 4.**
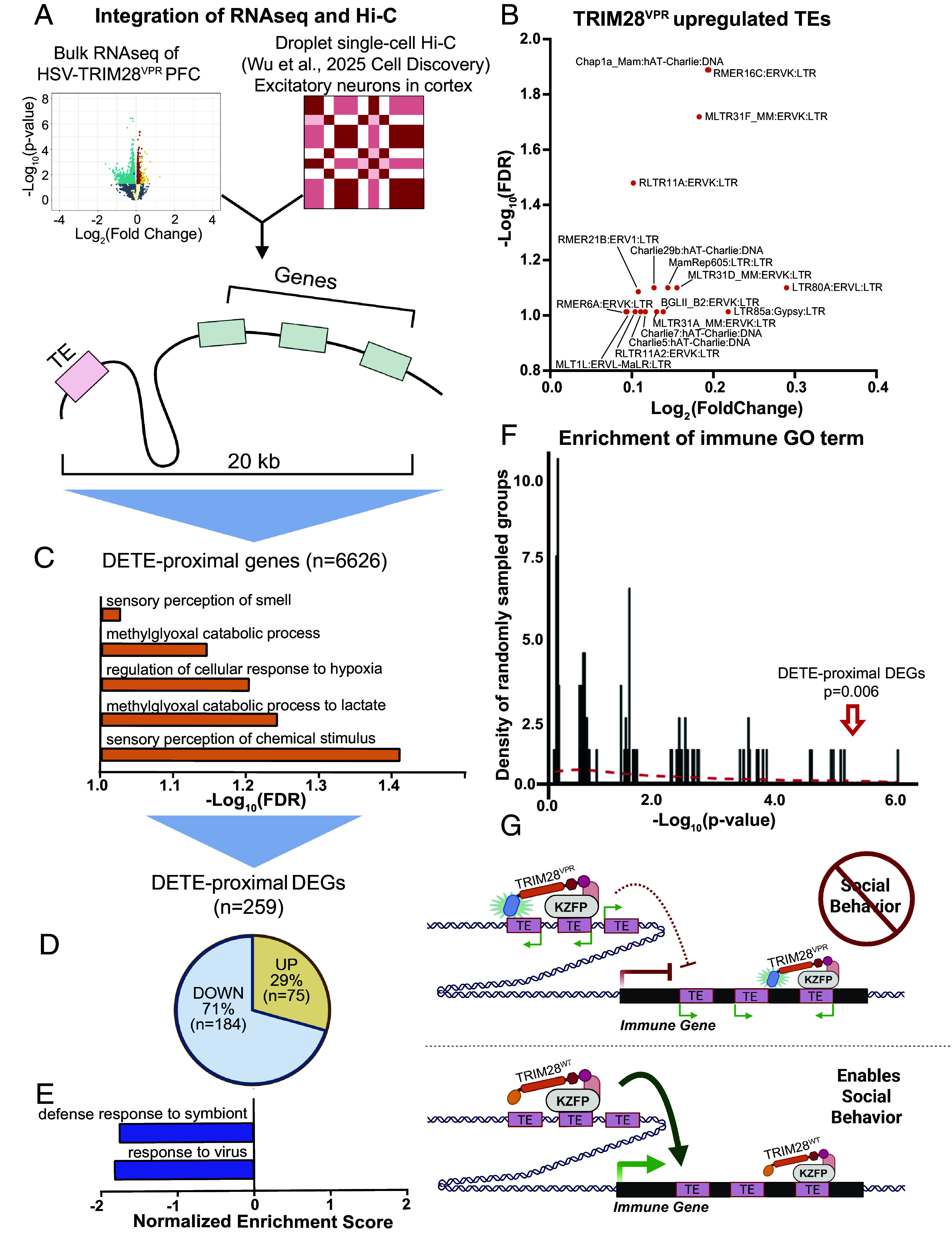
TEs released by TRIM28^VPR^ impair proximal immune gene expression. (*A*) Schematic of the integration of bulk RNAseq data from TRIM28^VPR^-treated mouse PFC and droplet single cell Hi-C analysis to determine genes in contact with DETE sites. A limit of 20 kb was used to identify contact pairs that are likely to represent *cis*-regulatory contacts. (*B*) TRIM28^VPR^ DETEs (all upregulated) that survive a significance cutoff of FDR < 0.1 (n = 17). (*C*) Overrepresentation analysis of gene ontology (GO) terms of all genes identified as forming contact pairs with a DETE within 20 kb (DETE-proximal genes, n = 6,626). Top five enriched GO terms are shown. (*D*) Breakdown of DETE-proximal DEGs (n = 259) by direction of regulation, demonstrating most DETE-proximal DEGs are downregulated (71%). (*E*) GSEA of DETE-proximal DEGs demonstrates enrichment for downregulated immune terms. All significant terms are shown. (*F*) Random sampling analysis of 100 subsets of n = 259 DETE-proximal genes for a representative significant immune GO term demonstrates that the DETE-proximal DEG subset is particularly enriched for immune-related genes (*P* = 0.006, percentile score = 1.98). (*G*) Cartoon depicting the proposed molecular mechanism linking PFC TRIM28 dysfunction and function to transposable elements, gene expression, and social behaviors. (*Top*) TRIM28^VPR^ activates the expression of TEs proximal to immune genes, which impedes immune gene expression and disrupts social behaviors. (*Bottom*) TRIM28^WT^ maintains transcriptional silencing of TEs, which enables immune gene expression and facilitates complex social behaviors.

Previous studies investigating chromatin looping suggest TE-derived *cis*-regulatory elements can exert transcriptional control on distal target genes made physically proximal in the 3D genome organization (44, 45). Other studies note such TE-derived enhancers can be located in intergenic or intronic TE-rich regions (46), suggesting that HSV-TRIM28^VPR^-mediated escape of such enhancer TEs may disrupt *cis*-regulation of target genes and causally contribute to the transcriptional divergence across TRIM28 variants. To mechanistically test this, we utilized publicly available droplet-based single cell Hi-C data (47) from 3-mo-old male and female mouse frontal cortices, specifically focusing on excitatory neurons, as this cell-type is manipulated by our neuron-targeting HSVs (Fig. 4*A*). We identified contact pairs containing TRIM28^VPR^ DETEs that survive a 10% FDR correction (Fig. 4*B*) and genes that occurred within a 20 kb proximity (called DETE-proximal genes). There were 6,626 DETE-proximal genes, and collectively, these genes were not particularly enriched for immune functions (Fig. 4*C*; gene list in Dataset S4, GO terms in Dataset S6). Of these DETE-proximal genes, 259 were significant DEGs in the PFC of TRIM28^VPR^-treated mice (called DETE-proximal DEGs) and these DEGs were primarily downregulated (Fig. 4*D*; gene list in Dataset S5). A GSEA revealed that these DETE-proximal DEGs were enriched for immune functions (Fig. 4*E*). To test whether TE escape preferentially interrupts proximal immune gene expression, we randomly sampled 100 subsets of 259 genes from the total 6,626 DETE-proximal genes and determined immune gene enrichment via a representative immune response GO term. We established an empirical distribution of the enrichment *P*-values and calculated the percentile of the p-value of our DETE-proximal DEGs in the distribution. We observed that the DETE-proximal DEGs were enriched for the immune GO term (*P*-value = 0.006) which is in the top 1.98 percentile of significance for this immune term relative to the randomly sampled DETE-proximal genes (Fig. 4*F*). We corroborated this finding in all TRIM28 datasets by using EnhancerAtlas (48) to identify that DETE-associated DEGs were particularly enriched for immune genes (*SI Appendix*, Fig. S5). These data establish a mechanistic link between TRIM28-regulated TEs and immune genes, wherein HSV-TRIM28^VPR^-driven TE release directly impedes the expression of physically proximal immune genes in contact pairs with TEs. We illustrate this mechanistic relationship between KZFP–TRIM28 gene regulatory control, genomic TEs, immune genes, and social behaviors in Fig. 4*G*.

### Repletion of PFC Immune Function Rescues TRIM28^VPR^-Driven Social Deficits.

**Fig. 5.**
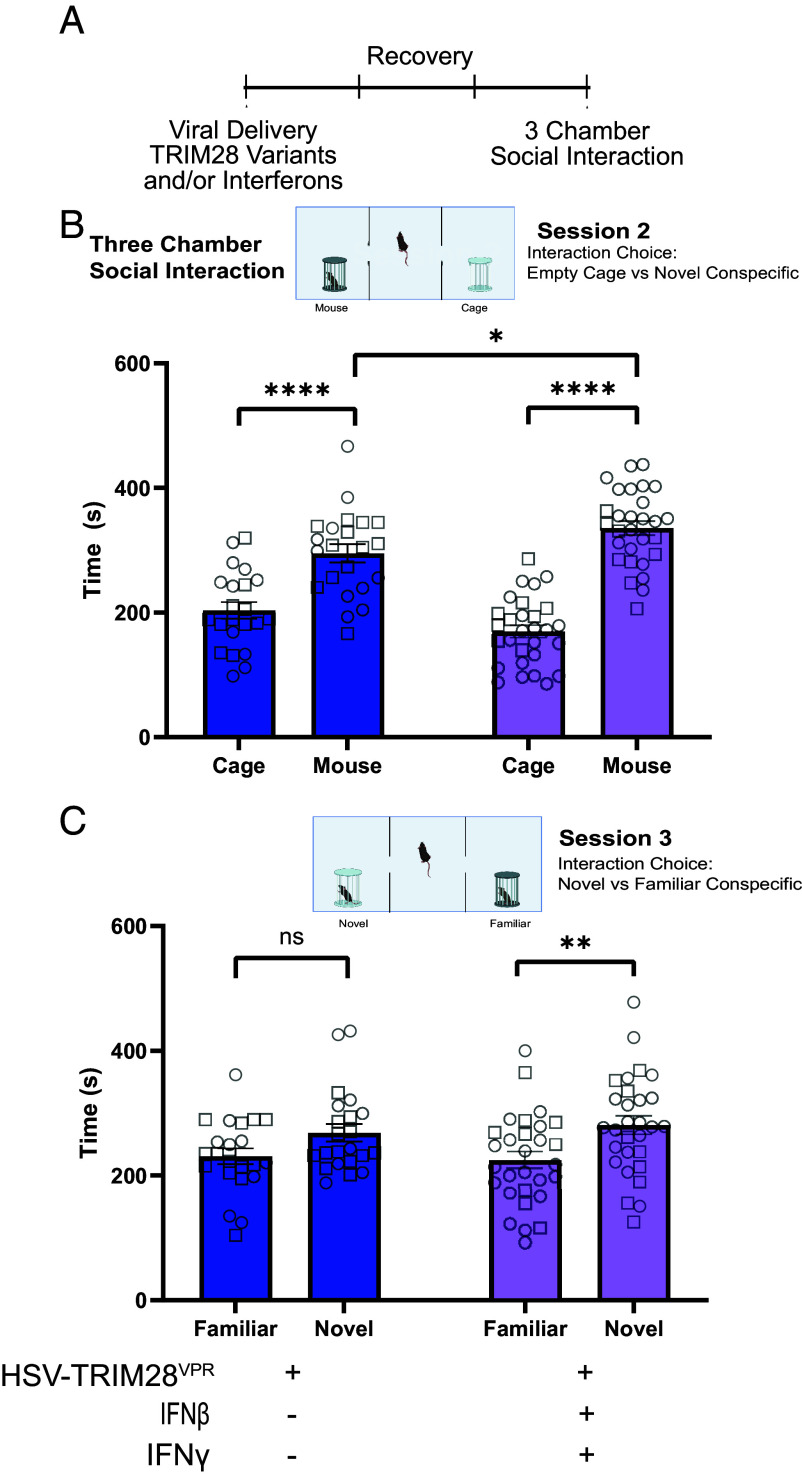
Repletion of PFC interferon function rescues TRIM28^VPR^-driven social deficits. (*A*) Timeline of viral delivery and three-chamber social Interaction testing. Each hash represents 1 d (HSV-TRIM28^VPR^ + VEH: n = 10 females, 12 males; HSV-TRIM28^VPR^ + IFN: n = 9 females, 20 males). (*B*) Coadministration of HSV-TRIM28^VPR^ with exogenous IFNβ and IFNγ increases time spent socializing with a novel age- and sex-matched conspecific in Session 2 of three-chamber social interaction (Interaction term *P* = 0.003; HSV-TRIM28^VPR^ + IFN “Mouse” v. HSV-TRIM28^VPR^ + VEH Mouse, *P* = 0.0409, two-way ANOVA with Bonferroni correction). Both HSV-TRIM28^VPR^ + IFN and HSV-TRIM28^VPR^ + VEH-treated groups displayed preference for social interaction over the empty cage (*P* < 0.0001, two-way ANOVA with Bonferroni correction). (*C*) In Session 3, HSV-TRIM28^VPR^ + VEH displayed a lack of preference for novel social interaction, while HSV-TRIM28^VPR^ + IFN restored preference for novel social interaction (Interaction term *P* = 0.5226; pairwise *P* > 0.05, = 0.0071, respectively, two-way ANOVA with Bonferroni correction). Squares indicate females; circles indicate males.

To determine whether the observed social deficits were causally produced by HSV-TRIM28^VPR^-driven deficiencies in PFC immune function, we tested whether treating HSV-TRIM28^VPR^ mice with exogenous interferon could restore normal social behavior. We codelivered HSV-TRIM28^VPR^ with a mixture of 10 ng each of interferon beta and gamma (IFN) or saline vehicle (VEH) and completed three-chamber social interaction on day four postsurgery, mirroring the timeline used in Figs. 2 and 5*A*. We observed that transduction with HSV-TRIM28^VPR^ + VEH recapitulated the social behavior deficit in session three as previously observed, with mice displaying no preference for novel social interaction (Fig. 5*B*, *P* > 0.05, two-way ANOVA with Bonferroni correction). However, HSV-TRIM28^VPR^ + IFN mice restored a preference for novel social interaction in this session (Fig. 5*C*, *P* = 0.0071, two-way ANOVA with Bonferroni correction). Interestingly, in session two, HSV-TRIM28^VPR^ + IFN mice displayed further increased sociability compared to HSV-TRIM28^VPR^ + VEH, spending increased total time spent socializing with the target mouse (Fig. 5*B*, *P* = 0.0409, two-way ANOVA with Bonferroni correction). Finally, we confirmed that administration of exogenous IFN did not impede HSV-mediated transduction of PFC neurons (*SI Appendix*, Fig. S6). These data indicate that disruption of endogenous immune functions is directly responsible for the observed HSV-TRIM28^VPR^-mediated social deficits and that these behavioral deficits are reversible by restoring interferon function in PFC.

## Discussion

Here, we demonstrate that TRIM28 functions in mouse PFC to repress and stabilize genomic TEs, enable immune gene expression, and facilitate complex social behaviors characterized by awareness and interest in social novelty. By inverting normal repressive TRIM28 function using synthetic HSV-TRIM28^VPR^, we demonstrated that mice developed social deficits characterized by loss of preference for novel social interaction in three-chamber social interaction and five-trial social memory tasks and a diminished capacity to engage in complex social hierarchies in the social dominance tube test (Fig. 2), without producing any nonsocial behavioral or cognitive deficits (*SI Appendix*, Figs. S2 and S3). We observed HSV-TRIM28^VPR^ caused substantial activation of TEs and downregulation of genes involved in immune functions, including response to type 1 and type 2 interferon (Fig. 3). These TRIM28-regulated TEs form proximal contacts with immune-related genes in 3D chromatin conformation, and the transcriptional escape of TEs via HSV-TRIM28^VPR^ impedes immune gene expression (Fig. 4). HSV-TRIM28^VPR^-driven social deficits were reversible by exogenous supplementation of interferon cytokines (Fig. 5), indicating that these social deficits were causally produced by impairments in PFC immune signaling.

These data are consistent with a growing body of literature linking social behaviors with brain adaptive immune functions (6–8, 23, 25, 49–56). Here, we find that KZFP/TRIM28-mediated genomic stability of cortical TEs is a key molecular mediator that links brain immune gene expression and complex prosocial behaviors. Further, the use of TRIM28 variants, including TRIM28^NFD^ which compete with endogenous TRIM28 function, suggests that TE silencing in this brain region is not solely established at singular developmental timepoint, but rather is constantly maintained by the persistent functions of TRIM28 and related molecular processes in the PFC. Thus, the conditions in which KZFPs, TRIM28, and TEs become dysregulated in the brain may contribute to our understanding of social dysfunction that characterizes many brain disorders.

Interestingly, we observed HSV-TRIM28^VPR^-mediated downregulation of immune genes is particularly driven by genes in proximal contact pairs with activated TEs (Fig. 4). Given the existing literature linking TE-rich genomic regions with *cis*-regulatory mechanisms (14–19), we posit that these TRIM28-controlled genomic loci may regulate immune genes, particularly interferons, through *cis*-regulatory mechanisms that require stabilization and repression of TEs. Notably, while a broad set of canonical genes exist in contact pairs with TRIM28^VPR^-dysregulated TEs, a disproportionately immune-enriched subset also appear as TRIM28^VPR^ DEGs, suggesting a targeted regulatory relationship between TEs and immune genes. This recapitulates existing literature suggesting that *cis*-regulatory mechanisms evolved by co-opting specifically intronic and intergenic TE-rich regions and further supports our hypothesized mechanism (Fig. 4*G*) (46).

While the immune-related DEG profile of HSV-TRIM28^VPR^- and HSV-TRIM28^NFD^-treated mice is consistent with a decrease in inflammatory signaling (Figs. 3 *I*–*K*, 4*E*, and *SI Appendix*, Figs. S4 and S5), the social behavioral profile is similar to that of a sickness-like or inflammation-induced phenotype, which is characterized by a decreased social novelty preference and a disruption to social hierarchy (57–59). However, mice lacking adaptive immunity (SCID mice) or the IFNγ gene also display a lack of social approach reversible by introduction of lymphocytes from IFNγ-competent mice (21), similar to how interferon repletion prevented the deficits induced by HSV-TRIM28^VPR^ (Fig. 5). This suggests that a balance of immune system activity is required to maintain a typical social behavioral profile.

All experiments presented in this study were completed with both male and female mice. In our behavioral studies, we initially included sex as a variable in multiway ANOVA statistical analysis to account for the possibility of detecting a sex difference in the behavioral consequences of dysregulation of TRIM28 transcription. However, we elected to combine all male and female data as there was no significant difference between sexes in any assay measured (Three-way ANOVA, main effect for sex *P* > 0.05; Dataset S7). The absence of a detectable sex difference in our social behavioral data supports the notion that TRIM28-mediated PFC molecular mechanisms similarly govern complex social behavior in both male and female mice, irrespective of well-noted baseline sex differences in the rodent social behaviors (60, 61).

Notably, chromatin modifications and TE dysregulation in the brain have been linked to ASD and ASD-like phenotypes in human populations and in animal models, particularly those with social behavior deficits (62–71). Heterozygous deletions or conditional knockouts of *TSHZ3*, a repressive zinc finger protein, in mice cause changes in social and repetitive behaviors similar to individuals with ASD (63, 69). In particular, *TSHZ3* conditional knockout in cortical projection neurons, such as those targeted by our PFC-delivered HSV, specifically impacts social, but not repetitive, behaviors (69). Additionally, the inhibition of brain chromatin-modifying enzymes such as HDACs has been shown to both induce and rescue social behavior deficits in different mouse models of ASD (62, 64–66). Further, prenatal exposure to the nonspecific HDAC inhibitor valproic acid causes the release of endogenous retroviruses, which persists across multiple generations of mice and is associated with social deficits that also persist across generations (65). TE transposition has been linked to social behaviors in canine studies, with the direction of effects dependent upon the genes affected by TE insertion (71). In several human sequencing studies, insertions of TEs have been linked to ASD, and these are particularly prevalent in intronic and intergenic regions, often enriched in known ASD risk genes (67, 68, 70).

Previously, our group has shown that a particular member of the KZFP family, ZFP189, modulates social behavior while tuning TE-dependent, immune-related neurobiological mechanisms (22). Here, we utilize novel TRIM28 variants to induce transcriptional dysregulation via the collective function of the KZFP family of proteins. We observed consistency in results, as inverting the function of either ZFP189 or TRIM28 induced social behavioral deficits while sparing nonsocial behaviors. This points to PFC KZFPs beyond just ZFP189 as involved in regulating social behaviors. Interestingly, these social deficits are nuanced, characterized by disruptions in the valence of social novelty and cooperative group-based behaviors, but not social recognition and memory, which suggests impact to higher-order, complex social cognition. The exact cognitive or motivational underpinnings, and the full resolution of these behavioral deficits, require future clarification.

In sum, we present a synthetic biology approach to collectively dysregulate the transcriptional control of the KZFP family of proteins within the PFC via re-engineering of their cognate binding partner TRIM28. In this manner, we observed that intra-PFC function of TRIM28 is necessary to maintain normal social behaviors, particularly higher-order social cognition involved in the salience of novel and group-based social interactions. Further, we showed that the gene transcriptional changes caused by inversion of TRIM28 transcriptional control are characterized by derepression of TEs and disruption of immune-related genes modulated by proximal TE-rich genomic regions. This work further clarifies the brain molecular link between PFC KZFP function, TE stability, and immune expression, all of which form a molecular regulatory axis to enable complex social behaviors necessary for group living.

## Materials and Methods

See the *SI Appendix* for a more detailed description of all methods.

### Subjects.

Male and female C57BL/6J mice (8 to 10 wk old) from Jackson Laboratories were used. Mice were group housed (5 mice/cage) on a 12-h light/dark cycle (lights on at 6 am/off at 6 pm) with ad libitum access to food and water. All mice were used in accordance with protocols approved by the Institutional Care and Use Committees at Virginia Commonwealth University School of Medicine.

### Viral Packaging.

We de novo synthesized TRIM28^NFD^, TRIM28^WT^, and TRIM28^VPR^ and subcloned each variant into HSV expression plasmids via ThermoFisher Scientific gateway LR Clonase II cloning reaction and Gateway LR Clonase II Enzyme mix kit (catalog number 11791-020 and 11971-100). Colonies were Maxi-prepped (Qiagen Cat # 12163) and shipped to the Gene Delivery Technology Core at Massachusetts General Hospital for HSV packaging. Once packaged, aliquots were made and stored in −80 °C to be used in viral gene transfer through stereotaxic surgery.

### Viral Gene Transfer.

Stereotaxic surgeries targeting the PFC were performed as previously described (31, 32). For the interferon repletion experiment, HSV-TRIM28^VPR^ was codelivered with a mixture of 10 ng each of interferon beta and gamma (Thermo Fisher) (IFN) or saline vehicle (VEH).

### N2a Cell Culture.

*Mus musculus* Neuro-2a (N2a; ATCC^®^ CCL-131™) neuroblast cell culture was grown and maintained as previously described (22).

### ZNF189RE- RenSP pLV[Exp]- EGFP:T2A:Puro Cloning and Lentivirus Packing.

The ZNF189 response element (RE)-basal TK promoter-RenSP luciferase gene from our previous ZNF189RE-RenSP luciferase reporter vector (22) was synthesized and subcloned into a Mammalian Gene Expression Lentiviral Vector (pLV[Exp]-EGFP:T2A:Puro) and packaged in lentivirus at a titer of 4.82 × 10^8^ TU/mL by VectorBuilder. The vector ID is VB230720-1406wne, which can be used to retrieve detailed information about the vector on vectorbuilder.com.

### ZNF189RE- RenSP-N2a Stable Cell Pool.

Low passage N2a cells (p5.5) were plated in a 6-well plate (3 × 10^5^ cells per well) and were grown until 70% confluent at the time of transduction. Following the Lentivirus In Vitro Applications User Instructions (Version 2.0, 2022-01-07) from VectorBuilder, cells were infected with the virus at multiplicity of infection (MOI) = 2, 3, and 5 in 1 mL of medium. When the vector EGFP expression was visible under fluorescence microscopy (Bio-Rad ZOE™ Fluorescent Cell Imager) from day 2 of postinfection, puromycin selection (1.5 μg/mL in 3 mL of medium per well) was applied for 4 d until uninfected cells were killed. Transduced cells with MOI of 2, 3, and 5 were mixed together as a ZNF189RE- RenSP-N2a stable pool and maintained in medium containing 2 μg/mL puromycin.

### Transfection and Luciferase Reporter Assay.

Using normal N2a cells, we applied the Effectene Transfection Reagent (Qiagen # 301427) to cotransfect the reporter plasmid DNA of ZNF189 RE (25 ng) with our synthetic TRIM28 variants and an unmodified ZFP189 expression plasmid DNA (100 to 120 ng with equal molar weight). In our RE-RenSP-N2a stable pool, we performed transfection only with TRIM28 variant expression vectors using the same transfection protocol. An unmodified GFP empty expression vector (p1005gw Δ*CCDB*) and an expression plasmid containing an untethered VPR domain were used as background controls.

On day 3 posttransfection, using a BMG Labtech POLARstar Omega Microplate Reader, the GFP Fluorescent (FI) was measured for equal transfection efficiency confirmation and normalization, followed by relative luminescence unit (RLU) using Renilla luciferase assay system (Promega, #E2820).

### Western Blot.

Western blots were performed in cultured N2a cells and in PFC tissue homogenate. Protein concentrations were determined using Pierce™ 660 nM Protein Assay Reagent (Thermo Fisher, #1861426). Membranes were probed with Trim28 antibody (Invitrogen, #MA5-32378, 1:2,000 dilution) followed by StarBright Blue 700 Goat Anti-Rabbit IgG (Bio-Rad, #12004161, 1:5,000 dilution), hFABTM Rhodamine Anti-Tubulin Primary Antibody (Bio-Rad, #12004165, 1:7,500 dilution), and hFABTM Rhodamine Anti-Actin Primary Antibody (Bio-Rad, #12004163, 1:5,000 dilution. Images were acquired by ChemidocMP Imaging System (Bio-Rad, #12003154).

### Behavioral Testing.

Behavioral analyses were performed automatically by video tracking software (Ethovision Noldus) (72). All behavioral tests were performed in a specified behavioral suite under red light.

### Three-Chamber Social Interaction Test.

The three-chamber test was used to assess sociability (session 2) and interest in social novelty or social discrimination (session 3). The test consisted of three 10-min sessions, beginning with a habituation session. Time spent in each chamber was recorded. Sociability was measured by comparing the time spent in the chamber with the novel mouse versus an empty cup. Social novelty was measured by comparing the time spent in the chamber with a novel versus familiar mouse.

### Five-Trial Social Memory Test.

Five-trial social memory was tested as previously described (22, 33) to determine ability to recognize novel versus familiar animals. Time spent in the interaction zone for the first minute of each trial was measured.

### Social Dominance Tube Test.

Animal social dominance was tested as previously described (22, 34, 35). For 5 d, baseline social hierarchy was determined by once-daily tube tests for all animals within a five-mouse cage in a randomized order. In each cage, the most dominant and most subordinate mice received intra-PFC HSV-TRIM28 variants, whereas the remaining cage-mates received intra-PFC HSV-GFP. For the following 5 d, the tube tests were repeated to determine postsurgery social hierarchy. Social dominance was measured by calculating the percentage of wins in the tube test (number of wins/number of tests × 100%).

### Elevated Plus Maze.

Elevated plus maze was performed according to established procedure (73). The percentage of time spent in the open arms was calculated (time spent in open arms/300 s) × 100% = % time spent in open arms).

### Novelty Suppressed Feeding.

Following 8 h of food restriction beginning at lights on, mice were individually placed in a large rat cage containing bedding and a single piece of normal chow for 10 min. Latency to feed was manually scored by a researcher blind to treatment.

### Novel Object Recognition Test.

Novel object recognition in the Y maze was tested as previously described (36, 37). Correct alternations in the Y maze were scored based on whether a mouse explored all three arms of the maze before repeating an arm. Novelty preference index was calculated on the second trial, defined as percent of time spent interacting with the novel object divided by total time spent interacting with either object.

### Sucrose Preference Test.

Sucrose preference test was performed as previously described (74). Percent sucrose preference is expressed as (Δ weight sucrose)/(Δ weight sucrose + Δ weight water) × 100%.

### Tissue Preparation and RNA Sequencing.

Mice virally manipulated with HSV-TRIM28^NFD^, -TRIM28^WT^, -TRIM28^VPR^, or HSV-GFP were used in RNAseq analysis. PFC tissue collection and RNA extraction, purification, and sequencing were performed as routinely done by our group (22, 75, 76).

### RNAseq Quality Control and Gene Quantification.

After quality control of raw FASTQ files, high-quality reads were aligned to the Mus musculus GRCm39 reference genome using STAR (version 2.7.11a) with the recommended parameters. We employed TEtranscripts (39) (version 1.09) in the quantification of both gene and TE expression levels by integrating genomic and RepeatMasker annotations (Hammell lab). After extraction of gene hit counts, the gene hit counts table was used for downstream differential expression analysis according to DESeq2’s standard pipeline. DEG lists were generated relative to the HSV-GFP virus condition, and all viral conditions were analyzed both separated by sex and with pooling across sexes. Volcano plots with DEGs and DETEs were assembled using ggplot in RStudio. Raw and processed RNAseq gene expression data are available via the Gene Expression Omnibus data (GSE294558).

### Genomic Annotation of TE and Potential Regulated Genes.

We annotated genomic features overlapping with the origins of DETEs generated from the above analysis.

### DEG Analysis Approach.

DEG tables from DESeq2 and annotated DETE data tables from the above analysis were imported into RStudio for further analysis. DETEs were categorized by class and plotted using the ggplot package. Rank–rank hypergeometric overlap testing was completed on unfiltered DEG tables with DETEs excluded using the RRHO2 package. GSEA was conducted using WebGestalt (77) with a range in category size from 5 to 300 genes and ontology terms were calculated regardless of FDR. Results were plotted as a volcano plot using ggplot, and a significance criterion of FDR < 0.05 was applied. DETEs were filtered for unique genomic origins and sorted by their predicted annotated genomic features, then plotted using the ggplot package. We compiled all DEGs with a known TE association, then applied an unadjusted *P* < 0.05 cutoff from expression data from our DESeq tables. DEGs associated with DETEs were analyzed using overrepresentation analysis using WebGestalt (77), and the gene ontology results were plotted using ggplot.

### Hi-C Analysis.

Droplet single cell Hi-C (dscHi-C) data from the 3-mo-old mouse cortex were obtained from Wu et al. (47) (GEO Accession GSM8709626). Adjusted *P*-values were calculated for TRIM28^VPR^-regulated DETEs. Using only contact pairs originating from excitatory neurons, we identified contacts between DETE genomic locations and canonical genes within 20 kb (DETE-proximal genes, Dataset S4) and those within 20 kb that appeared in the TRIM28^VPR^ DEG list DETE-proximal DEGS, Dataset S5). Gene ontology or pathway enrichment analysis was performed (Dataset S6). Random sampling of 100 subsets from the total list of DETE-proximal genes was performed, and the enrichment *P*-value of the GO term “leukocyte activation involved in immune response” was determined for each subset, generating an empirical distribution with which to compare the DETE-proximal DEGs.

### Statistical Analysis.

The three-way ANOVA tests on social behavioral data were conducted using the rstatix package in R, and simple main effects were reported. Due to the lack of variance as a function of biological sex, all behavioral data were condensed to combine male and female data for further analysis. All data were otherwise analyzed in GraphPad Prism 10. In all figures, results were expressed as mean ± SE (SEM). Statistical analysis outputs from Prism are available in Dataset S7.

## Supplementary Material

Appendix 01 (PDF)

Dataset S01 (XLSX)

Dataset S02 (XLSX)

Dataset S03 (XLSX)

Dataset S04 (XLSX)

Dataset S05 (XLSX)

Dataset S06 (XLSX)

Dataset S07 (XLSX)

## Data Availability

RNA sequencing data have been deposited in GEO Omnibus (GSE294558). Previously published data were used for this work (47).
