# Supplementary material for: Derepression of transposable elements in the mouse prefrontal cortex disrupts social behavior
Source: Proc Natl Acad Sci U S A. 2025 Dec 17;122(51):e2510663122. doi: 10.1073/pnas.2510663122 (PMC12745702; doi:10.1073/pnas.2510663122)
Supplement: Supplementary file 1 — Appendix 01 (PDF) [file pnas.2510663122.sapp.pdf]

SUPPLEMENTARY INFORMATION

“Derepression of transposable elements in mouse prefrontal cortex disrupts social behavior”

Kim, Smith, et al.

Materials and Methods

Subjects

Male and female C57BL/6 J mice (8 to 10 weeks old) from Jackson Laboratories were used. Mice were group housed (5 mice/cage) on a 12-hour light/dark cycle (lights on at 6am/off at 6 pm) with ad libitum access to food and water. All mice were used in accordance with protocols approved by the Institutional Care and Use Committees at Virginia Commonwealth University School of Medicine.

Viral packaging

We *de novo* synthesized TRIM28^{NFD}, TRIM28^{WT}, and TRIM28^{VPR} and sub-cloned each variant into HSV expression plasmids via ThermoFisher Scientific gateway LR Clonase II cloning reaction and Gateway LR Clonase II Enzyme mix kit (catalog number 11791-020 and 11971-100). Colonies were Maxi-prepped (Qiagen Cat # 12163) and shipped to the Gene Delivery Technology Core at Massachusetts General Hospital for HSV packaging. Once packaged, aliquots were made and stored in −80 °C to be used in viral gene transfer through stereotaxic surgery.

Viral gene transfer

Stereotaxic surgeries targeting the PFC were performed as previously described[31,32]. Mice were anesthetized with I.P. injection of ketamine (100 mg/kg) and xylazine (10 mg/kg) dissolved in sterile saline solution. Mice were then placed in a small-animal stereotaxic device (Kopf Instruments) and the skull surface was exposed. 33-gauge needles (Hamilton) were utilized to infuse 1.0 µL of virus at a rate of 0.2 µL/min followed by a 5-min rest period to prevent backflow. For the interferon repletion experiment, HSV-TRIM28^{VPR} was co-delivered with a mixture of 10 ng each of interferon beta and gamma (Thermo Fisher) (IFN) or saline vehicle (VEH). The

following coordinates were used to target the PFC: Bregma: anterior-posterior: +1.85 mm, medial-lateral ±0.75 mm, dorsal-ventral −2.5 mm, 15° angle.

N2a Cell culture

Mus musculus Neuro-2a (N2a; ATCC® CCL-131™) neuroblast cell culture were maintained in 1:1 EMEM enriched / EMEM growth medium mixture (Quality Biological, #112-039-101; ATCC, #30 2003 or Corning, #10-009-CV) with 5% FBS (HyClone, #SH30071.03IH30-45) and 100 U/ml Penicillin Streptomycin (Gibco, #15140122) in a 37 °C and 5% CO_2 Thermo Scientific HERAcell-150i CO_2 incubator. N2a cells were split twice weekly at 1:6 up to passage ~50 as previously described[22].

ZNF189RE- RenSP pLV[Exp]- EGFP:T2A:Puro Cloning and lentivirus packing

The ZNF189 response element (RE)-basal TK promoter-RenSP luciferase gene from our previous ZNF189RE-RenSP luciferase reporter vector[22] was synthesized and subcloned into a Mammalian Gene Expression Lentiviral Vector **(**pLV[Exp]-EGFP:T2A:Puro) and packaged in lentivirus at a titer of 4.82 x10^8 TU/ml by VectorBuilder. The vector ID is VB230720-1406wne, which can be used to retrieve detailed information about the vector on vectorbuilder.com.

ZNF189RE- RenSP-N2a stable cell pool

Low passage N2a cells (p5.5) were plated in a 6-well plate (3x10^5 cells per well) and were grown until 70% confluent at the time of transduction. Following the Lentivirus In Vitro Applications User Instructions (Version 2.0, 2022-01-07) from VectorBuilder, cells were infected with the virus at multiplicity of infection (MOI) = 2, 3, and 5 in 1ml of medium. When the vector EGFP expression was visible under a fluorescence microscopy (Bio-Rad ZOE™ Fluorescent Cell Imager) from day 2 of post-infection, puromycin selection (1.5ug/ml in 3ml of medium per

well) was applied for 4 days until uninfected cells were killed. Transduced cells with MOI of 2, 3, and 5 were mixed together as a ZNF189RE- RenSP-N2a stable pool and maintained in medium containing 2ug/ml puromycin.

Transfection and luciferase reporter assay

The day before transfection, approximately 1.5-2x10^4 cells in 100ul medium per well (N2a or RE-RenSP-N2a stable pool) were seeded into 96-well clear bottom microplates with white walls (Corning, # 3610). Wells reached roughly 80-90% confluent in 24 hours. Using normal N2a cells, we applied the Effectene Transfection Reagent (Qiagen # 301427) to co-transfect the reporter plasmid DNA of ZNF189 RE (25ng) with our synthetic TRIM28 variants and an unmodified ZFP189 expression plasmid DNA (100-120ng with equal molar weight). In our RE-RenSP-N2a stable pool, we performed transfection only with TRIM28 variant expression vectors using the same transfection protocol. An unmodified GFP empty expression vector (p1005gw $\Delta \boldsymbol{CCDB}$) and an expression plasmid containing an untethered VPR domain were used as background controls.

On day 3 post-transfection, using a BMG Labtech POLARstar Omega Microplate Reader, the GFP Fluorescent (FI) was measured for equal transfection efficiency confirmation and normalization, followed by relative luminescence unit (RLU) using Renilla luciferase assay system (Promega, #E2820). Data was analyzed using GraphPad Prism 10 software.

Western blot

For cell culture samples, N2a cells (4x10^4 cells in 1.5mL medium per well) were seeded in 6-well plate (Corning, #3516) and co-transfected the reporter plasmid DNA of ZNF189 RE (100ng) with our synthetic TRIM28 variants (400ng) using the Effectene Transfection Reagent (Qiagen, #301427). On day 2 post-transfection, cells were collected and separated into nuclear and

cytoplasmic fractions using NE-PER Nuclear and Cytoplasmic Extraction Reagents (Thermo Fisher, #78833). After protein concentration were determined using Pierce™ 660nM Protein Assay Reagent (Thermo Fisher, #1861426), 18.75 µg of protein from three biological replicates of cytoplasmic fractions was loaded onto a 7.5% Tris-Glycine gel (Biorad, # 5678024) and then separated through SDS-PAGE. After the transfer onto PVDF membrane, the membranes were blocked for one hour with 5% BSA in 0.1%Tween/1xTBST. Membranes were probed with Trim28 antibody (Invitrogen, #MA5-32378, 1:2000 dilution) for one hour at RT followed by StarBright Blue 700 Goat Anti-Rabbit IgG (Biorad, #12004161, 1:5000 dilution), hFABTM Rhodamine Anti-Tubulin Primary Antibody (Biorad, #12004165, 1:7500 dilution) and hFABTM Rhodamine Anti-Actin Primary Antibody (Biorad, #12004163, 1:5000 dilution) for one hour at RT. Images were acquired by ChemidocMP Imaging System (Biorad, #12003154). Data are representative of three independent experiments.

For tissue samples, TRIM28 variant-manipulated PFC tissue punches were collected (see "Tissue Preparation and RNA Sequencing" section below for further details). Tissue punches were lysed in Pierce™ RIPA buffer (Thermo Fisher, #89901) and protein concentrations were determined as above. Each well was loaded with 39 µg of protein homogenate, which was separated and blocked as above. Membranes were probed with Trim28 antibody (Invitrogen, #MA5-32378, 1:2000 dilution) overnight at 4ºC, followed by the same secondary antibody protocol as above.

Behavioral testing

Behavioral analyses were performed automatically by video tracking software (Ethovision Noldus)[72]. All behavioral tests were performed in a specified behavioral suite under red light.

Three chamber social interaction test

The three chamber test was used to assess sociability and interest in social novelty or social discrimination. The testing arena consisted of three adjacent chambers (each 41 × 21 × 41 cm) separated by two clear plastic dividers and connected by open doorways (5 × 9 cm). The test consisted of three 10-min sessions. The subject mouse begins each session in the middle chamber. In the first session, subject mice were habituated to the arena and allowed free investigation of the three chambers. In the subsequent sociability session, a novel C57BL/6J sex- and age-matched conspecific (target mouse 1) was placed in a cylindrical cage (20 cm height × 10 cm diameter solid bottom; with clear bars spaced 2 cm apart) in one of the side chambers and an identical cage was placed in the opposite side chamber. In the social novelty session, the empty cage was replaced by target mouse 1 in a fresh cage. A second novel C57BL/6J sex- and age-matched conspecific (target mouse 2) was placed at the previous position of target mouse 1 in a new cylindrical cage. The chamber and cylindrical cages were thoroughly cleaned with 70% EtOH between animals and before the first animal. Time spent in each chamber was recorded. Sociability was measured by comparing the time spent in the chamber with the novel mouse vs. an empty cup. Social novelty was measured by comparing the time spent in the chamber with a novel vs. familiar mouse.

Five-trial social memory test

Five-trial social memory was tested as previously described[22,33] to determine ability to recognize novel versus familiar animals. Subject mice were placed into the open arena (43 × 43 × 43 cm) with an empty wire cage (10 × 5 × 30 cm) at one side (interaction zone) and given 2.5 min of habituation to explore the arena before being returned to their home cage. A novel C57BL/6J sex and age-matched conspecific was then placed within the wire cage (interaction zone), and subject mice were placed back into the open arena for four subsequent 2.5 min trials with a 10 min inter-trial interval. In trial 5, a novel C57BL/6J sex and age-matched conspecific was

placed within the interaction cage to measure dishabituation. Time spent in the interaction zone for the first minute of each trial was measured.

Social dominance tube test

Animal social dominance was tested as previously described[22,34,35] in a transparent Plexiglas tube measuring 30.5 cm in length and 3 cm diameter, a size sufficient to permit one subject mouse to pass through without being able to reverse direction. The tube was set on a plastic table in the designated behavioral suite and trials were manually recorded by a researcher blind to experimental groups. Animals were placed at opposite ends of the tube and released. A subject was declared the “winner” when its opponent backed out of the tube, with all 4 paws outside of the tube. The maximum test time allowed was 2 min. For 5 days, baseline social hierarchy was determined by once-daily tube tests for all animals within a five-mouse cage in a randomized order. In each cage, the most dominant and most subordinate mice received intra-PFC HSV-TRIM28 variants whereas the remaining cage-mates received intra-PFC HSV-GFP. For the following 5 days, the tube tests were repeated to determine post-surgery social hierarchy. Social dominance was measured by calculating the percentage of wins in the tube test (number of wins/number of tests x 100%).

Elevated plus maze

The EPM apparatus is constructed of black Plexiglas and consists of two open arms (33 × 6 cm) and two closed arms (33 × 9.5 × 20 cm) facing connected by a central platform (5 × 7 cm)[73]. The maze was elevated 63 cm above the floor. C57BL/6J mice (31 mice across two cohorts following 3 chamber social interaction testing, and 29 mice across two cohorts with no prior behavioral test) were placed individually in the right-side closed arm facing the center of the plus-maze. Placement of all four paws into an arm was registered as an entry in the respective arm. The time spent in each arm and total locomotion was recorded during the 5 min EPM test.

The platform of the maze was cleaned with 70% EtOH following each trial and before the first trial. The percentage of time spent in the open arms was calculated (time spent in open arms/300 s) x 100% = % time spent in open arms).

Novelty suppressed feeding

The novelty suppressed feeding arena consisted of a large rat cage (30 x 19 x 39 cm) containing woodchip bedding and a single pellet of normal chow. Following 8 hours of food restriction beginning at lights on, mice were individually placed in the arena for 10 minutes. Latency to feed was manually scored by a researcher blind to treatment. If a test mouse did not approach the food and begin to feed by the end of the 10-minute trial, the mouse was returned to its home cage, and a latency of 10 minutes was recorded.

Novel object recognition test

Novel object recognition was tested as previously described[36,37]. 24 hours prior to the test day, subject mice were allowed to freely explore and habituate to the Y-maze arena for 10 minutes. Correct alternations were scored based on whether a mouse explored all three arms of the maze before repeating an arm. On the test day, subject mice underwent two test trials with a 10-minute inter-trial interval. The first trial presents subject mice with two identical object copies in two arms of the Y-maze arena. Following 5 minutes of exploration, subject mice were returned to their home cage. During the inter-trial interval, the arena and both interaction objects were thoroughly cleaned with 70% EtOH, and one object was replaced with a second, completely different object. Subject mice were returned to the arena and again allowed to explore both objects. The arena and all interaction objects were thoroughly cleaned with 70% EtOH between each subject mouse and before the first trial of the day. Novelty preference index was calculated on the second trial, defined as percent of time spent interacting with the novel object divided by total time spent interacting with either object.

Sucrose preference test

Sucrose preference test was performed as previously described[74]. Following the novel object recognition test, mice were single housed with *ad libitum* access to food and two bottles: one with water and one with 1% w/v sucrose solution. For four days, liquid intake was measured daily by weighing the bottles, and the left/right positions of the bottles were swapped to avoid position preferences. Percent sucrose preference is expressed as (Δ weight sucrose)/(Δ weight sucrose + Δ weight water) × 100%.

Tissue preparation and RNA sequencing

Mice virally manipulated with HSV-TRIM28^{NFD}, -TRIM28^{WT}, -TRIM28^{VPR}, or HSV-GFP were used in RNAseq analysis. Mice were cervically dislocated and decapitated without anesthesia, and the brains were removed and sectioned into 1 mm coronal slices using brain matrices. Central tissue punch containing bilateral PFC (12 gauge; internal diameter, 2.16 mm) were snap frozen on dry ice and stored at -80 °C, as is routinely performed by our group[22,75,76]. RNA was extracted and purified using RNeasy (Qiagen, Hilden, Germany), and total RNA was quantified with the Qubit RNA HS Assay Kit (Thermo Fisher Scientific, Waltham, MA). RNA quality control assays were performed on the TapeStation 4200 (Agilent, Santa Clara, CA), and the average RNA integrity number for all samples exceeded 8.6. Ribosomal RNA depletion and library preparation (Illumina Ribo-Zero) was performed, and RNAseq was carried out at Genewiz with the following configuration: 2 × 150 paired-end reads on an Illumina (San Diego, CA) sequencing platform (HiSeq 2500) with a sequencing depth of ~52 million reads per sample (mean = 52 ± 0.45 million). Other overall sample sequencing statistics include the mean quality score (37.77 ± 0.05) and the percent of bases ≥ 30 (92.20 ± 0.19).

RNAseq quality control and gene quantification

Raw RNAseq FASTQ files were subjected to quality control using FastQC (version 0.11.9) to assess read quality. Adapter sequences were removed using Trimmomatic (version 0.39) with further trimming off low-quality bases (16 bases and 3 bases from the head and tail, respectively). We also dropped the 4-base window of average sequencing quality < 15 and finally the reads less than 50 bases. After the quality control, high-quality reads were aligned to the Mus musculus GRCm39 reference genome using STAR (version 2.7.11a) with the recommended parameters. We employed TEtranscripts[39] (version 1.09) in the quantification of both gene and TE expression levels by integrating genomic and RepeatMasker annotations (Hammell lab). After extraction of gene hit counts, the gene hit counts table was used for downstream differential expression analysis.

The RNAseq count data were normalized, and dispersion estimates were obtained according to DESeq2's standard pipeline. DEG lists were generated relative to the HSV-GFP virus condition, and all viral conditions were analyzed both separated by sex and with pooling across sexes. Wald tests were performed to determine differentially expressed genes (DEGs) and differentially expressed transposable elements (DETEs). Gene and TE IDs with p-values < 0.05 were considered DEGs and DETEs respectively. Volcano plots with these DEGs and DETEs were assembled using ggplot in Rstudio. Raw and processed RNAseq gene expression data are available via the Gene Expression Omnibus data (GSE294558).

Genomic annotation of TE and potential regulated genes

We annotated genomic features overlapping with the origins of DETEs in generated from the above analysis. The genomic features included in this analysis were the promoter, enhancer, histone H3K4 tri-methylation and CTCF binding site revealed by ENCODE project, as well as the exon, intron, UTR from GRCm39 gene annotations. For the overlapping enhancers, we

obtained their potential regulated genes predicted using ChIP-seq data from EnhancerAtlas v2.0[48].

DEG analysis approach

DEG tables from DESeq2 and annotated DETE data tables from the above analysis were imported into Rstudio for further analysis. DETEs were categorized by class and plotted using the ggplot package. Rank-rank hypergeometric overlap testing was completed on unfiltered DEG tables with DETEs excluded using the RRHO2 package. GSEA analysis was conducted using WebGestalt[77] with a range in category size from 5-300 genes and ontology terms were calculated regardless of FDR. Results were plotted as a volcano plot using ggplot, and a significance criteria of FDR < 0.05 was applied. DETEs were filtered for unique genomic origins and sorted by their predicted annotated genomic features, then plotted using the ggplot package. We compiled all DEGs with a known TE association, then applied an unadjusted $p < 0.05$ cutoff from expression data from our DESeq tables. DEGs associated with DETEs were analyzed using over-representation analysis using WebGestalt[77], and the gene ontology results were plotted using ggplot.

Hi-C analysis

Droplet single cell Hi-C (dscHi-C) data from 3 month old mouse cortex was obtained from Wu et al. (*Cell Reports* 2025)[47] (GEO Accession GSM8709626). Adjusted p-values were calculated for TRIM28^{VPR}-regulated DETEs, resulting in 17 DETEs at a 10% FDR cutoff, and we extracted the genomic locations of these DETEs and lifted them from GRCm39 coordinates to mm10 coordinates, as this is the reference genome utilized in the dscHi-C dataset. Using only contact pairs originating from excitatory neurons, we identified contacts between DETE genomic locations and canonical genes, limiting the flanking region size to 20kb to capture close range contacts more likely to represent *cis*-regulatory relationships (Supp. Dataset 4). These DETE-

proximal genes (n = 6626) were filtered according to whether they appeared in the DEG list for TRIM28^{VPR}, resulting in n = 259 DETE-proximal DEGs (Supp. Dataset 5). Gene ontology or pathway enrichment analysis was performed using clusterProfiler for the list of all DETE-proximal genes (Supp. Dataset 6) and using WebGestalt[77] for DETE-proximal DEGs. Random sampling of 100 subsets of n = 259 genes from the total list of n = 6626 DETE-proximal genes was performed, and the enrichment p-value of the GO term "leukocyte activation involved in immune response" was determined for each subset, generating an empirical distribution with which to compare the DETE-proximal DEGs.

Statistical analysis

The three-way ANOVA tests on social behavioral data were conducted using the rstatix package in R, and simple main effects were reported. Due to the lack of variance as a function of biological sex, all behavioral data was condensed to combine male and female data for further analysis. All data was otherwise analyzed in GraphPad Prism 10. In all figures, results were expressed as mean ± standard error (S.E.M.). *In vitro* validation data was analyzed by one-way ANOVA with Bonferroni correction (Fig. 1c, e). Behavioral data was analyzed as two-way ANOVA with Bonferroni correction (Fig. 2b-c, e-g, j-k: Fig. 5a-b), one-way ANOVA with Bonferroni correction (Supp. Fig. 2b-d, f, h) and Wilcoxon signed rank test (Fig. 2j-k). The distribution of DETEs by class was compared by Chi-square goodness of fit test (Fig. 3d). *P*-value < 0.05 was considered statistically significant. Experimental sample sizes were guided by power analyses and previously published experiments from our group and others. Animals were randomly assigned to viral treatment conditions. Experimenters were blind to treatment and experimental analysis was performed by automated Ethovision software. Statistical analysis outputs from Prism are available in Supplementary Dataset 7.

Supplementary Figures

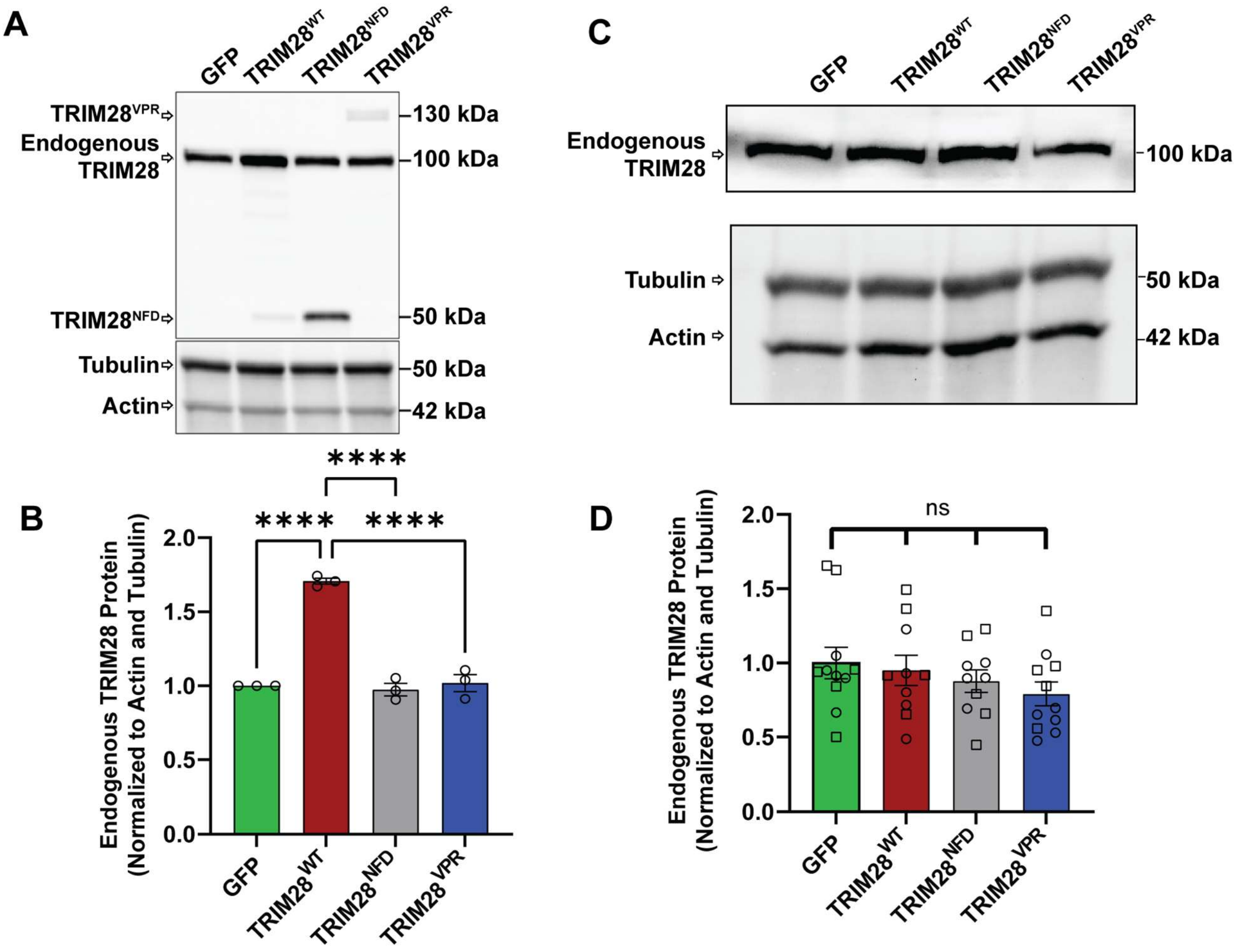

Supplementary Figure 1: TRIM28 variants do not alter endogenous TRIM28 expression. a) Representative image of western blot for TRIM28 protein in N2a cultured cells transfected with GFP or TRIM28 variant plasmids. $TRIM28^{WT}$ and endogenous TRIM28 runs at the expected molecular weight of 100 kDa, while $TRIM28^{NFD}$ runs at 50 kDa, and $TRIM28^{VPR}$ runs at 130 kDa. b) Quantification of TRIM28 band (100 kDa) in each variant, normalized to tubulin and actin housekeeping genes. TRIM28 is expressed at a significantly higher quantity in the $TRIM28^{WT}$ group than in the GFP, $TRIM28^{NFD}$, and $TRIM28^{VPR}$ groups (n = 3 per group, $p < 0.0001$ for all comparisons, one-way ANOVA with Bonferroni correction). c) Representative image of western blot for TRIM28 protein in microdissected, HSV-transfected PFC tissue punch. d) Quantification of TRIM28 band (100 kDa) in each variant condition, normalized to actin and tubulin housekeeping genes. Endogenous TRIM28 expression is not significantly different between conditions (n = 3 per group, $p > 0.05$, one-way ANOVA with Bonferroni correction). Squares indicate females; circles indicate males.

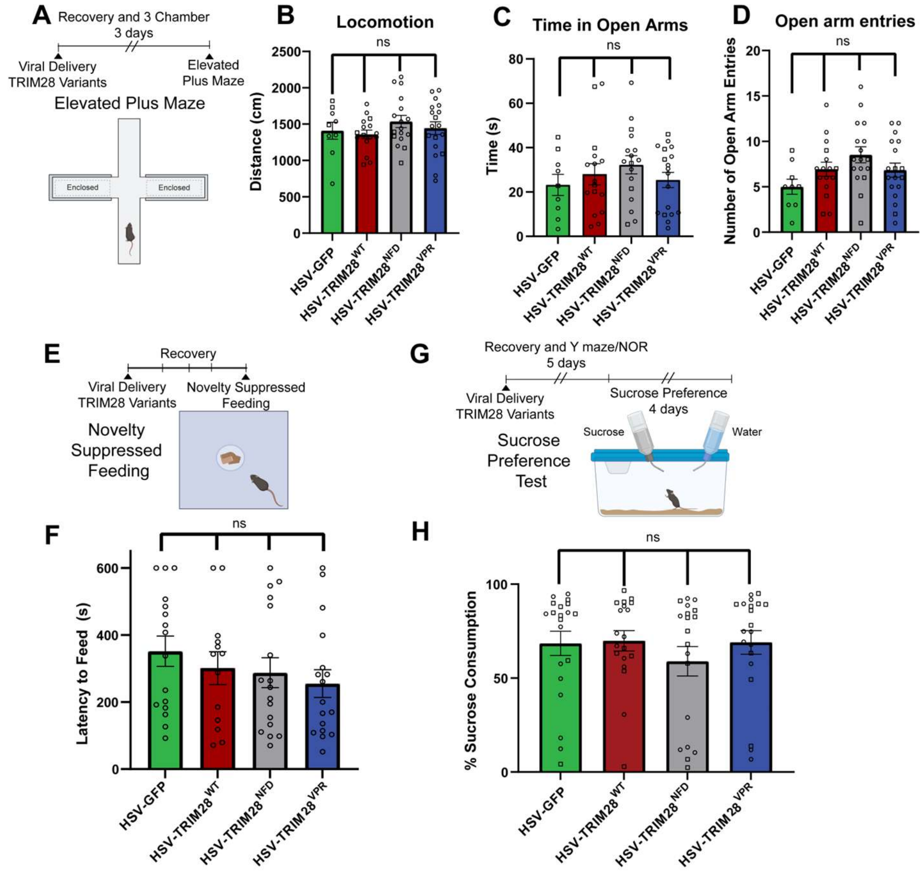

Supplementary Figure 2: Disrupting PFC TRIM28 function does not impact basal or anxiety-like behaviors. a) Timeline and schematic of the elevated plus maze (EPM) (HSV-GFP: n = 6 males, 3 females; HSV-TRIM28^{WT}: n = 12 males, 4 females; HSV-TRIM28^{NFD}: n = 13 males, 4 females; HSV-TRIM28^{VPR}: n = 14 males, 4 females). b-d) Locomotion, time spent in the open arms, and entries into the open arms of the EPM is not significantly impacted by TRIM28 variants ($p > 0.05$, one-way ANOVA with Bonferroni correction). e) Timeline and schematic of the novelty suppressed feeding arena (HSV-GFP: n = 15 males, HSV-TRIM28^{WT}: n = 13 males, HSV-TRIM28^{NFD}: n = 17 males, HSV-TRIM28^{VPR}: n = 17 males). f) There is no significant difference in latency to feed by viral treatment group ($p > 0.05$ for all, one-way ANOVA with Bonferroni correction). g) Timeline and schematic of the sucrose preference test (HSV-GFP: n = 10 females, 10 males; HSV-TRIM28^{WT}: n = 10 females, 9 males; HSV-TRIM28^{NFD}: n = 10 females, 10 males; HSV-TRIM28^{VPR}: n = 10 females, 10 males) h) Viral treatment group does not significantly impact the percentage of sucrose consumed throughout the testing period ($p > 0.05$ for all, one-way ANOVA with Bonferroni correction). Squares indicate females; circles indicate males. NOR: Novel Object Recognition.

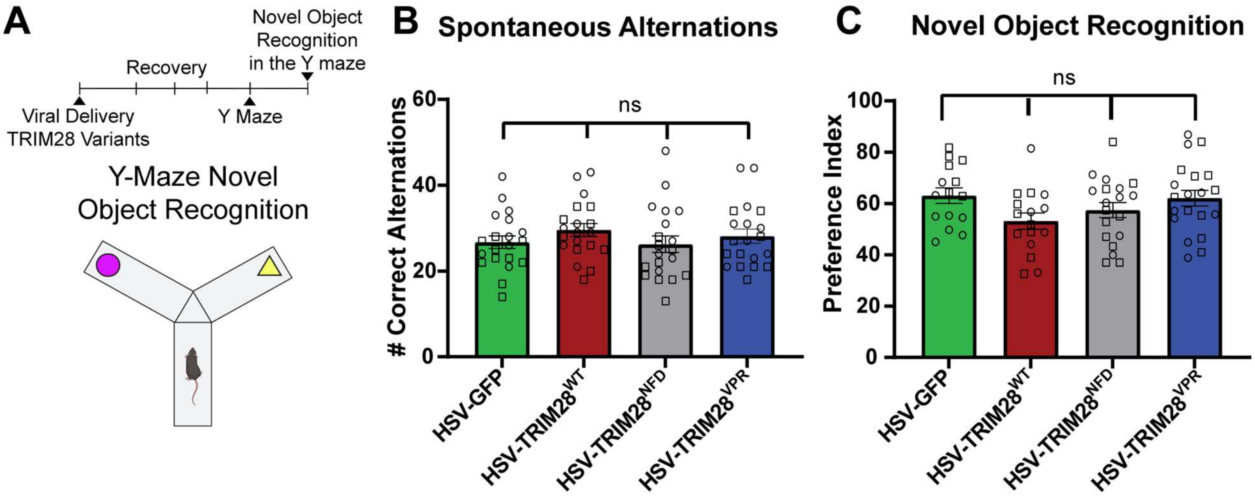

Supplementary Figure 3: Disrupting PFC TRIM28 function does not affect non-social object recognition. a) Timeline and schematic of novel object recognition in the Y-Maze (HSV-GFP: n = 10 females, 10 males; HSV-TRIM28^{WT}: n = 10 females, 9 males; HSV-TRIM28^{NFD}: n = 10 females, 10 males; HSV-TRIM28^{VPR}: n = 10 females, 10 males). b-c) Infusion of HSV-GFP or an HSV-TRIM28 variant to the prefrontal cortex (PFC) does not produce significantly different effects on the number of correct alternations or novelty recognition in the Y-Maze ($p > 0.05$ for all, one-way ANOVA with Bonferroni correction). Squares indicate females; circles indicate males.

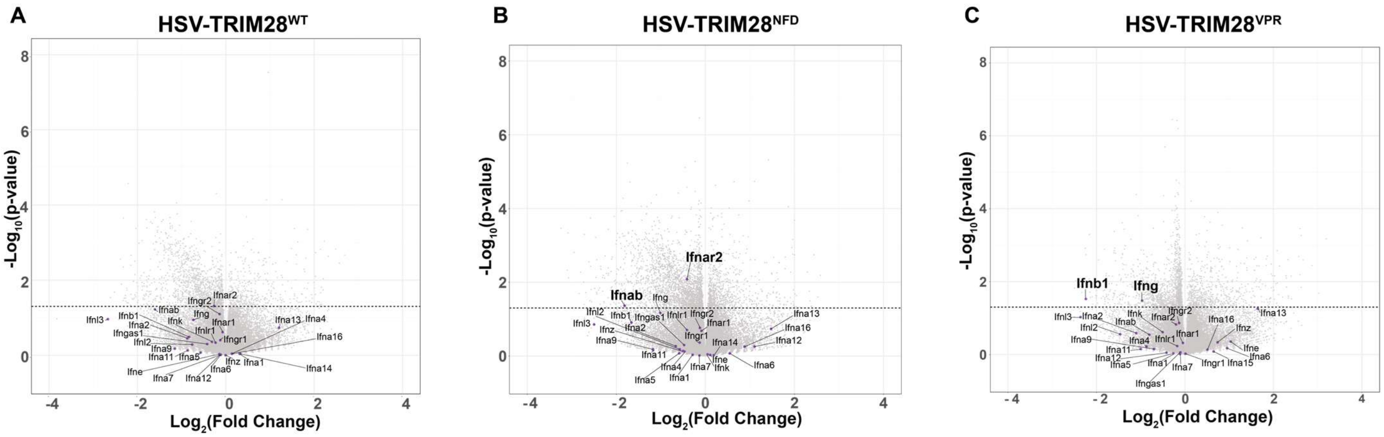

Supplementary Figure 4: Downregulation of interferon cytokines by synthetic TRIM28 variants. a-c) Volcano plots showing expression of interferon genes across HSV-TRIM28^{WT}, -TRIM28^{NFD}, and -TRIM28^{VPR} conditions. No interferon cytokines are differentially expressed in HSV-TRIM28^{WT}. Only interferon alpha-B is downregulated in HSV-TRIM28^{NFD}. Both interferon beta and gamma are downregulated in HSV-TRIM28^{VPR}.

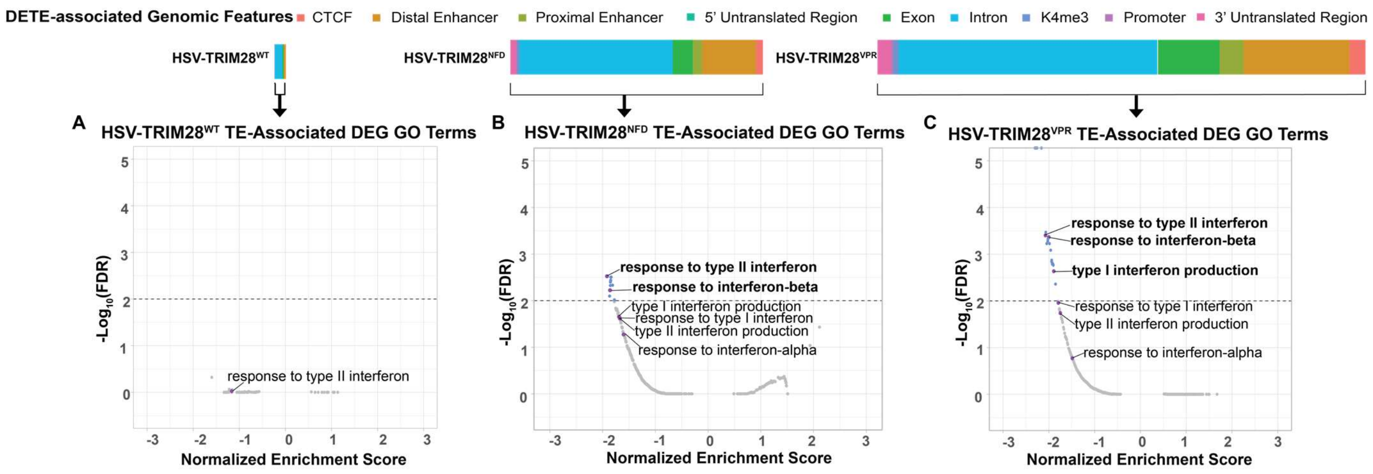

Supplementary Figure 5: TE-associated DEGs are enriched for immune genes. TE-associated DEGs were generated from searching DETEs through EnhancerAtlas and compiling a list of all significant DEGs associated with DETEs in each TRIM28 variant condition. a-c) Gene Set Enrichment Analysis was conducted on these TE-associated DEGs. Interferon related ontology terms are downregulated by HSV-TRIM28^{VPR} to a greater extent than HSV-TRIM28^{NFD}. A significance criterion of FDR < 0.01 was applied.

Representative Sample Brain

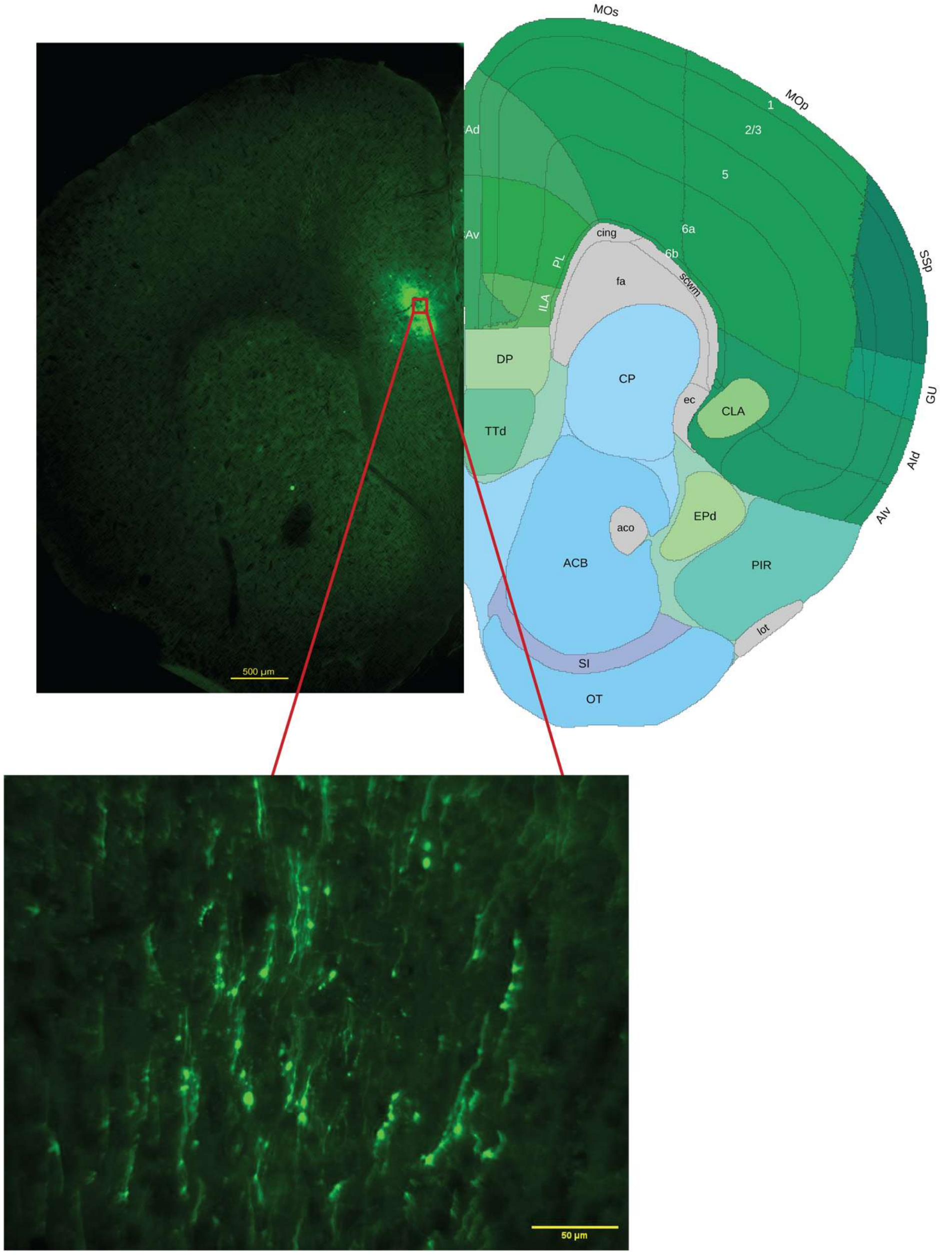

Supplementary Figure 6: HSV transduction is not impaired by administration of exogenous interferon. Co-delivery of HSV vectors with 10 ng each of interferon beta and gamma does not prevent viral transduction of PFC neurons. Representative PFC image shown.

**TE class**	**HSV-GFP vs HSV-TRIM28^{WT}**	**HSV-GFP vs HSV-TRIM28^{NFD}**	**HSV-GFP vs HSV-TRIM28^{VPR}**
**Unclassified**	3	8	7
**Non-LTR**	8	67	166
**LTR**	36	127	229
**DNA**	14	57	108
**Total**	61	259	510

Supplementary Table 1: Synthetic TRIM28 variants disrupt expression of all classes of transposable elements. HSV-TRIM28^{NFD} reflects derepression of TEs, while HSV-TRIM28^{VPR} reflects active transcription of TEs.

Genomic Feature	HSV-GFP vs HSV-TRIM28^{WT}	HSV-GFP vs HSV-TRIM28^{NFD}	HSV-GFP vs HSV-TRIM28^{VPR}
**CTCF**	80	4006	9057
**K4m3**	35	1562	3041
**Distal Enhancer**	1016	30637	60594
**Proximal Enhancer**	142	5359	13611
**Exon**	692	15950	35137
**5' Untranslated Region**	11	219	477
**Intron**	4393	87325	147483
**Promoter**	16	579	1410
**3' Untranslated Region**	99	3136	7681
**Total**	6484	148773	278491

Supplementary Table 2: Synthetic TRIM28 variants potentiate expression of transposable elements associated with distal enhancer and intronic genomic features. The most abundant genomic origin associated with DETEs are distal enhancer and intronic regions.

Titles and Legends for Supplementary Datasets

Supplementary Dataset 1: DESeq output of TRIM28^{WT} v. GFP comparison. Gene identifiers and expression changes in TRIM28^{WT}-treated mice compared to GFP-treated controls.

Supplementary Dataset 2: DESeq output of TRIM28^{NFD} v. GFP comparison. Gene identifiers and expression changes in TRIM28^{NFD}-treated mice compared to GFP-treated controls.

Supplementary Dataset 3: DESeq output of TRIM28^{VPR} v. GFP comparison. Gene identifiers and expression changes in TRIM28^{VPR}-treated mice compared to GFP-treated controls.

Supplementary Dataset 4: DETE-proximal genes. Mapped locations of TRIM28^{VPR}-dysregulated differentially expressed transposable elements (DETEs) and the genes that are mapped to a contact point within 20 kb.

Supplementary Dataset 5: DETE-proximal DEGs. List of TRIM28^{VPR} differentially expressed genes (DEGs) that are in contact pairs with a TRIM28^{VPR} differentially expressed transposable element (DETE) within 20 kb.

Supplementary Dataset 6: Biological Process Gene Ontology terms of all DETE-proximal genes. List of Gene Ontology (GO) terms from the PANTHER Biological Processes database of all DETE-proximal genes.

Supplementary Dataset 7: Statistical Analysis. Outputs from GraphPad Prism for statistical tests for all biochemical and behavioral data, by figure.
